# Prospective electrocardiographic and cardiovascular magnetic resonance alterations in the UK Biobank coronavirus disease 2019 repeat imaging study

**DOI:** 10.1016/j.jocmr.2025.101957

**Published:** 2025-09-10

**Authors:** Sucharitha Chadalavada, Ahmed Salih, Hafiz Naderi, Elisa Rauseo, Jackie Cooper, Stefan van Duijvenboden, Anwar A. Chahal, Gaith S. Dabbagh, Liliana Szabo, Mohammed Y. Khanji, Jose D. Vargas, Mihir Sanghvi, Kenneth Fung, Jose Paiva, Stefan K. Piechnik, Betty Raman, Patricia B. Munroe, Aaron Mark Lee, Alborz Amir-Khalili, Luca Biasiolli, John P. Greenwood, Paul M. Matthews, Wenjia Bai, Stefan Neubauer, Nay Aung, Nicholas C. Harvey, Zahra Raisi-Estabragh, Steffen E. Petersen

**Affiliations:** a William Harvey Research Institute, NIHR Barts Biomedical Research Centre, Queen Mary University of London, Charterhouse Square, London, UK; b Barts Heart Centre, St Bartholomew’s Hospital, Barts Health NHS Trust, West Smithfield, London, UK; c Department of Population Health Sciences, University of Leicester, Leicester, UK; d Department of Computer Science, University of Zakho, Zakho, Kurdistan, Iraq; e Division of Cardiovascular Medicine, Radcliffe Department of Medicine, University of Oxford, National Institute for Health Research Oxford Biomedical Research Centre, Oxford University Hospitals NHS Foundation Trust, Oxford OX3 9DU, UK; f Big Data Institute, La Ka Shing Centre for Health Information and Discovery, University of Oxford, Oxford, UK; g Cardiac Electrophysiology Section, Division of Cardiovascular Diseases, University of Pennsylvania, Philadelphia, Pennsylvania, USA; h Department of Cardiovascular Diseases, Mayo Clinic, Rochester, Minnesota, USA; i Center for Inherited Cardiovascular Diseases, WellSpan Health, Lancaster, Pennsylvania, USA; j Semmelweis University, Heart and Vascular Center, Budapest, Hungary; k Department of Cardiology, US Department of Veterans Affair Medical Center, Washington, District of Columbia, USA; l Circle Cardiovascular Imaging Inc., Calgary, Alberta, Canada; m Leeds Institute of Cardiovascular & Metabolic Medicine, University of Leeds, and Leeds Teaching Hospitals NHS Trust, Leeds, UK; n UK DRI Centre and Department of Brain Sciences, Imperial College London, London, UK; o Department of Computing, Imperial College London, London, UK; p MRC Lifecourse Epidemiology Centre, University of Southampton, Southampton SO16 6YD, UK; q NIHR Southampton Biomedical Research Centre, University of Southampton and University Hospital Southampton NHS Foundation Trust, Southampton, UK

**Keywords:** SARS-COV-2, Cardiac magnetic resonance, Electrocardiogram, Cardiovascular disease, Long COVID, Myocarditis

## Abstract

**Background:**

Cardiovascular magnetic resonance (CMR) and electrocardiographic (ECG) abnormalities after coronavirus disease 2019 (COVID-19) are widely reported. However, the absence of pre-infection assessments limits causal inference from these studies. This study aims to compare interval change in CMR and ECG measures in participants with incident COVID-19 and matched uninfected controls in UK Biobank.

**Methods:**

UK Biobank participants with documented COVID-19 who had CMR and ECG performed before the pandemic were invited for repeat assessment, along with uninfected participants matched on age, sex, ethnicity, location, and date of baseline imaging. Automated pipelines were used to extract ECG phenotypes and CMR measures of cardiac structure and function, aortic distensibility, aortic flow, and myocardial native T1. Logistic regression was used to examine associations of baseline metrics with incident COVID-19. Standardized residual approach was used to compare the degree of interval change in CMR and ECG metrics between cases and controls.

**Results:**

We analyzed 2092 participants (1079 cases and 1013 controls) with average age of 60 ± 7 years. 47.1% were male. There was 3.2 ± 1.5 years between pre- and post-infection assessments. 3.6% of cases were hospitalized. Lower baseline left ventricular ejection fraction and worse longitudinal, circumferential, and radial strain were associated with higher risk of incident COVID-19. There were no significant differences in interval change of any CMR or ECG metric between cases and controls.

**Conclusion:**

While pre-existing cardiovascular abnormalities are linked to higher risk of COVID-19, exposure to infection does not alter interval change of highly sensitive CMR and ECG indicators of cardiovascular health.

## Graphical abstract

**Figure:**
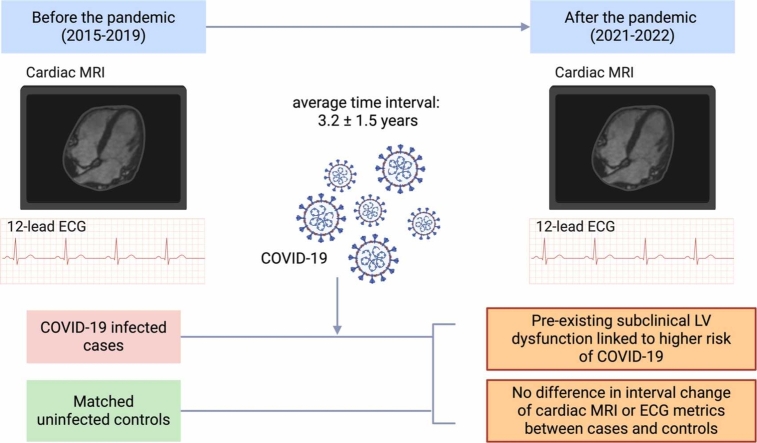
Created using Biorendr.com.

## 1 Introduction

Coronavirus disease 2019 (COVID-19), the illness caused by severe acute respiratory syndrome coronavirus 2 (SARS-COV-2) infection, has emerged as a major cause of morbidity and mortality worldwide [1].

While SARS-COV-2 primarily targets the respiratory system, its cardiovascular manifestations during acute infection are widely recognized and linked with poorer outcomes [2], [3], [4]. Biologic studies suggest distinct mechanistic drivers of cardiac involvement, including direct viral cardiotoxicity, immune dysfunction, and prothrombotic phenomena [5]. Furthermore, large-scale epidemiologic studies report elevated long-term cardiovascular risk many months after recovery from the acute illness [6], [7], [8].

Cardiovascular magnetic resonance (CMR) is the reference modality for assessing cardiac structure and function and uniquely permits noninvasive evaluation of myocardial tissue character [9]. International guidelines recognize CMR's utility for assessing cardiovascular involvement in COVID-19 [10], [11].

A number of studies have suggested persistent cardiac involvement after apparent recovery from COVID-19, based on abnormalities detected on CMR scans performed after infection [12], [13], [14]. However, the absence of CMR imaging before infection severely limits causal inference from these analyses, as it is not possible to distinguish pre-existing cardiovascular abnormalities from those that may have been caused by subsequent infection exposure. These considerations are particularly pertinent given that adverse cardiometabolic profile and pre-existing cardiovascular diseases are associated with both higher risk of COVID-19 and adverse CMR alterations [15]. Thus, while existing literature raises important questions about the long-term cardiovascular consequences of SARS-COV-2 infection, these are based on study designs with inherently high risk of confounding and reverse causation.

The UK Biobank COVID-19 Repeat Imaging Study was established to facilitate research in understanding the multiorgan impact of COVID-19, while addressing the outlined shortcomings in existing literature. Participants who had completed CMR imaging shortly before the pandemic as part of the UK Biobank Imaging Study and who had documented SARS-COV-2 infection were invited to have a repeat CMR scan. Repeat scanning was also performed, in the same way, for an equal number of matched uninfected participants. Thereby creating an internationally unique dataset with paired pre- and post-infection CMR imaging performed using standardized methods for confirmed cases and matched uninfected controls.

In the present study, we used electrocardiographic (ECG) and CMR data from the UK Biobank COVID-19 Repeat Imaging Study to evaluate potential causal relationships between SARS-COV-2 and cardiovascular health, considering: (1) differences in baseline ECG and CMR phenotypes of cases and controls; (2) association of baseline ECG and CMR phenotypes with incident COVID-19; (3) differences in the degree of interval change in ECG and CMR phenotypes before and after the pandemic in infected individuals and matched uninfected controls.

## 2 Methods

### 2.1 Study population and setting

The UK Biobank is a prospective cohort study including over half a million people recruited between 2006 and 2010 from various urban and rural settings across the UK. Individuals aged 40 to 69 living within 25 miles of 1 of 22 assessment centers were identified from National Health Service records and invited to participate. Participants who could not consent or complete baseline assessment due to ill health or discomfort were not recruited. There was no requirement for healthy status. Baseline assessment comprised highly detailed characterization of participant socio-demographics, lifestyle, and medical history, as well as a series of physical measures, and blood sampling [16]. The UK Biobank Imaging Study, which includes CMR, launched in 2015 and aims to scan 100,000 of the original participants. Extensive health record linkage is established for the entire UK Biobank cohort with hospital episode statistics (HES), Office for National Statistics death registration, and SARS-COV-2 test results from Public Health England.

The UK Biobank COVID-19 Repeat Imaging Study comprises a unique dataset of individuals with multiorgan imaging before and after SARS-COV-2 infection, and an equal number of matched uninfected controls [17]. Participants who had completed the imaging study before the pandemic and had a record of SARS-COV-2 infection were invited for repeat imaging (January 2021–February 2022). SARS-COV-2 infection status was ascertained from antigen (swab) test results, linked health records, and lateral flow antibody tests sent to participants [17]. Participants who had completed the imaging study before the pandemic (2015–2019), but who had no record of SARS-COV-2 infection in any of the linked data sources, were considered as potential controls. The present study analysis includes all UK Biobank COVID-19 Repeat Imaging Study participants (n = 2092) with at least one ECG or CMR available. The exclusion criteria were assessed on quality control protocols on a metric-by-metric basis and are detailed below.

### 2.2 Ethical approval

This study complies with the Declaration of Helsinki; the work was covered by the ethical approval for UK Biobank studies from the NHS National Research Ethics Service on June 17, 2011 (Ref 11/NW/0382) and extended on June 18, 2021 (Ref 21/NW/0157) with written informed consent obtained from all participants.

### 2.3 CMR image acquisition

CMR imaging was performed in dedicated centers, using standardized equipment and pre-defined acquisition protocols [18]. CMR scans are with 1.5T scanners (MAGNETOM Aera, Syngo Platform VD13A, Siemens Healthineers, Erlangen, Germany). These were research without any clinical indication. The acquisition protocol is detailed in a separate publication [18]. In brief, cardiac function was assessed using standard long and short cines performed using balanced steady state free precession (bSSFP) sequences. Myocardial native T1 mapping was performed in a single mid-ventricular short-axis slice using Shortened Modified Look-Locker Inversion recovery (ShMOLLI, WIP780B) sequences. Aortic compliance was derived from a transverse bSSFP cine at the level of the pulmonary trunk and right pulmonary artery. A phase contrast sequence is planned on both sagittal and coronal left ventricular (LV) outflow tract cines to capture aortic flow. The standard velocity encoding was set at 2 m/s and adjusted upward as needed.

### 2.4 CMR image analysis

**Fig. 1:**
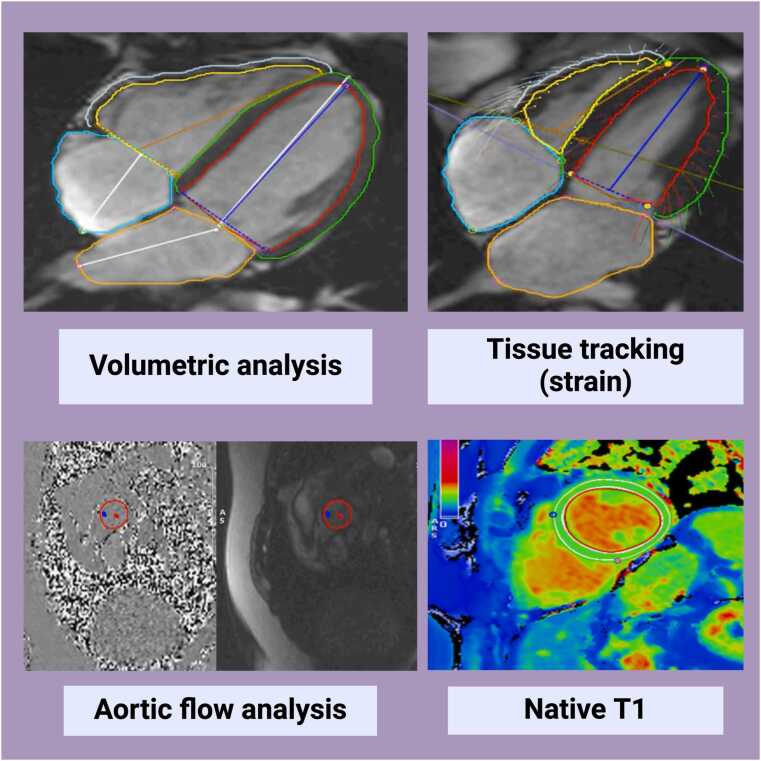
CMR image analysis. *CMR* cardiovascular magnetic resonance. Created on Biorendr.com

CMR scans were analyzed to derive volumetric quantification of all four cardiac chambers, feature tracking strain metrics from the left and right ventricles, aortic flow measurements, and global myocardial native T1 values (see Fig. 1). Circle Inc. CVI42 (Circle Cardiovascular Imaging, Calgary, Alberta, Canada) prototype 5.14.1.2875 batch processing was used for the segmentation of cardiac contours and aortic phase contrast images, as well as for native T1 data extraction. Circle Inc. CVI42 (prototype 5.13.7) was used for batch strain analysis. CVI42 image analysis tools are available as commercial products that have regulatory approval. The ability to analyze scans in large “batches” is the unique feature of the prototypes used in this study. The entire short-axis stack was used for volumetric assessment with simplified endocardial contour without papillary muscle detection. Volumetric and strain analysis excluded any short-axis slices with open contours. A 10% offset of epicardial and endocardial borders was used for the analysis of ShMOLLi (native T1) images. The full details of analysis settings and methods are presented in Supplementary Table 1. Aortic areas were derived from transverse cine images of the aorta using an automated tool previously developed and validated in the UK Biobank [19]. Aortic distensibility and strain were calculated using formulas detailed in Supplementary Table 2.

Statistical outliers and non-sensical data removal were applied as quality control measures for the whole dataset with the process validated with visual quality control, as described in a dedicated publication using a large subset of this UK Biobank COVID-19 Repeat Imaging Study [20]. This study demonstrates that when using the CVI42 batch processing pipelines combined with statistical outlier removal for chamber volumetric data, strain, native T1, and aortic flow data, the results are not different from those obtained following expert visual quality control and removal of poor-quality images/segmentations.

Supplementary Table 3 details the complete list of metrics derived, the quality control parameters set, and the number of cases included for each metric after applying the quality control criteria.

### 2.5 ECG analysis

All participants had a 12-lead ECG recorded alongside the pre- and post-pandemic imaging visits. Electrodes were placed in standard positions, recorded at a frequency of 500 Hz for 10 s (Cardiosoft v6.51 GE, Waukesha, Wisconsin), and stored in XML file format. These files were downloaded and reprocessed using GE MUSE v9.0 SP4, Marquette 12 SL [21]. The raw ECG signals were analyzed, and 31 ECG phenotypes were automatically extracted. We included only independent ECG leads (I, II, V1-6) as these are acquired directly. The global ECG biomarkers used are detailed in Supplementary Table 4 and definitions in Supplementary Table 5. The number of participants with ECG data analyzed after applying the quality control is detailed in Supplementary Table 6.

### 2.6 Participant characteristics

Age was taken as recorded at the pre-pandemic imaging visit. Sex, ethnicity, smoking, and alcohol were from self-report. Body mass index (BMI) was calculated from physical measurements taken at baseline imaging. Diabetes, hypercholesterolemia, and hypertension were defined using a combination of self-report, medication history, and HES records. The presence of pre-existing major cardiovascular diseases (myocardial infarction, heart failure, non-ischemic cardiomyopathies, valvular heart disease) was defined using self-report and HES records. The baseline use of cardiac medications (betablockers, statins, angiotensin converting enzyme inhibitors, antiplatelets, anticoagulants) was defined from self-report. Hospitalization due to COVID-19 was ascertained from HES records. The definitions and UK Biobank field IDs used for these variables are detailed in Supplementary Table 7.

### 2.7 Statistical analysis

Statistical analysis was performed using Python 3.9.7 software. The labels for cases and controls and matching on age, sex, ethnicity, location, and date of baseline imaging were performed centrally by UK Biobank.

Baseline characteristics are presented as number (percentage) for categorical variables, mean (standard deviation, SD) for normally distributed continuous variables, and median [interquartile range, IQR] for non-normally distributed continuous variables. The distribution of CMR and ECG metrics was assessed using the “skew” function in Python.

First, baseline CMR and ECG metrics of infected and uninfected participants were compared using independent t-test or Mann-Whitney test according to data distribution.

Second, the association of baseline CMR phenotypes with incident COVID-19 was examined using logistic regression, with SARS-COV-2 infection status (case vs control) set as the outcome and each CMR or ECG metric set as the exposure of interest, with adjustment for age, sex, ethnicity, deprivation, BMI, smoking, diabetes, hypertension, high cholesterol, and prevalent myocardial infarction.

Third, to elucidate whether exposure to SARS-COV-2 infection alters the trajectory of cardiovascular ECG and CMR phenotypic alterations, we tested the difference in change in CMR and ECG metrics between infected cases and matched uninfected controls. We calculated interval change for CMR and ECG measures from the baseline (pre-pandemic) and repeat visits for all participants. The first step involves regressing values from the baseline visit to calculate the predicted value for each CMR metric. We then compare to this the actual values for each CMR metric extracted from the repeat scans. This comparison is represented by calculated standardized residuals, which act as standardized change scores. These standardized change scores represent variation in the degree of change in each metric from that expected (predicted values) based on the initial imaging visit. Consideration of the baseline value in these estimates removes artifactual phenomena such as regression to the mean. This method is the established best practice for evaluating true differences in interval change of observational data and presents greater rigor compared to simple subtraction of measurements [22]. We thus compared average standardized risk scores for each metric between the infected and control group using an independent t-test or Mann-Whitney test according to data distribution.

A sub-analysis was performed in the subset of 34 cases with a record of COVID-19 hospitalization and their uninfected comparators propensity matched on age and sex. T-test analysis of the relevant CMR metrics at baseline and repeat imaging was performed to assess differential relationships in participants with severe SARS-COV-2 infection. If there were any significant results, then the second and third analyses described above would also be performed.

## 3 Results

**Fig. 2:**
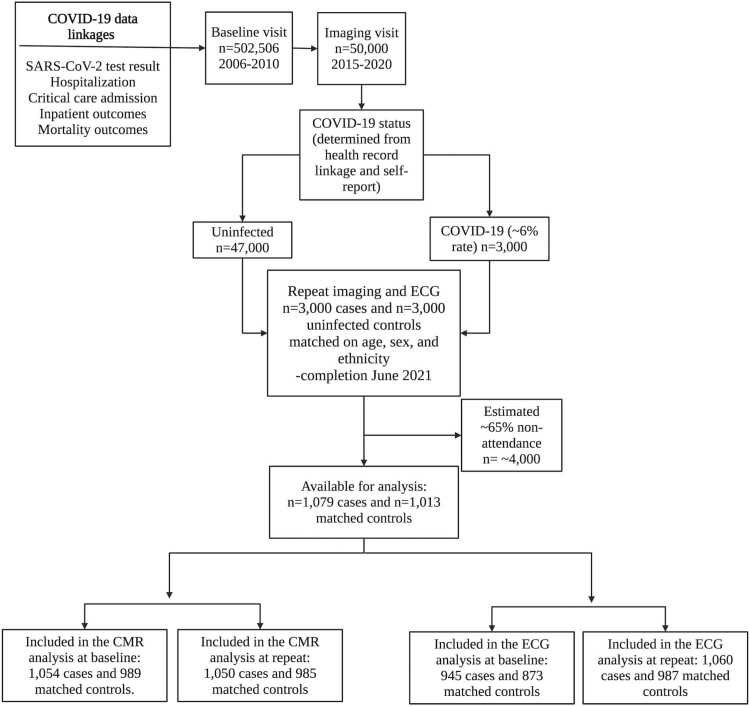
Timeline and number of participants included in the UK Biobank COVID-19 repeat imaging and ECG study. *COVID-19* coronavirus disease 2019, *SARS-CoV-2* severe acute respiratory syndrome coronavirus 2, *CMR* cardiovascular magnetic resonance, *ECG* electrocardiogram. Created using BioRendr.com

Among individuals who had completed a CMR scan as part of the UK Biobank Imaging Study before the pandemic, 2092 were recruited to the COVID-19 Repeat Imaging Study (Fig. 2). The cohort included 1079 participants with record of SARS-COV-2 infection (cases) and 1013 matched uninfected controls [17]. The mean interval between baseline and repeat imaging was 3.2 years (SD = 1.5). The median interval from March 2020 (initial COVID-19 outbreak in UK) to repeat scan was 1.3 years. For analyses using baseline CMR and ECG data, all participants with data available for that time point were included. Participants with pre- and post-pandemic ECG or CMR were included for analyses of interval change in CMR and ECG metrics.

Overall, 2043 participants were included in the analysis of baseline CMR metrics (n = 1054 cases and n = 989 controls) and 2035 participants for the repeat CMR metrics (n = 1050 cases and n =985 controls). Regarding the ECG data, the number of participants with available ECG biomarkers included in the analysis was 1818 at baseline (n = 945 cases and n = 873 controls) and 2047 at repeat visit (n = 1060 cases and n = 987 controls).

### 3.1 Baseline demographic and morbidity profile

The mean age was 60 years (SD of 7.5 for control and 7.7 for cases) in both cases and controls, and the sex and ethnicity distribution across the two cohorts was similar, indicating satisfactory matching of these variables (Table 1). Over 95% of participants were of White ethnicity. Among those with a record of SARS-COV-2 infection, 4% (n = 38) had a record of hospitalization.

**Table 1:** Study population participants’ characteristics at baseline imaging.

	Control (n = 989)	COVID cohort (n = 1054)
Demographics		
Age at baseline imaging (y), mean (SD)	60.1 (7.5)	60.1 (7.7)
Sex (male (female))	47.1% (52.9%)	45.8% (54.2%)
White, n (%)	923 (95.5)%	983 (95.2)%
BAME, n (%)	42 [4.5%]	51 [4.8%]
Townsend deprivation score, mean (SD)	−1.70 (2.73)	−1.46 (2.86)
Current smoking n (%)	28 (2.9%)	37 (3.6%)
BMI, median, kg/m^2^, (IQR)	25.8 [23.1, 28.8]	26.1[23.6, 29.2]
Hypertension, n (%)	203 (21.2%)	238 (23.1%)
Diabetes, n (%)	43 (4.5%)	50 (4.9%)
Myocardial infarction, n (%)	17 (1.8%)	21 (2.0%)
Heart failure	3 (0.3%)	3 (0.3%)
Non-ischemic cardiomyopathy	1 (0.1%)	1 (0.1%)
Valvular heart disease	10 (1.0%)	14 (1.3%)
Hospitalized for COVID-19	-	38 (3.6%)
Betablockers	34 (3.4%)	36 (3.4%)
Angiotensin converting enzymes or angiotensin receptor blockers	85 (8.6%)	94 (8.9%)
Statins	119 (12.0%)	149 (14.1%)
Antiplatelets	10 (1.0%)	11 (1.0%)
Anticoagulants	2 (0.20%)	3 (0.3%)
Any cardiovascular medication	176 (17.8%)	201 (19.0%)

*BAME* Black, Asian and Minority Ethnic, *BMI* body mass index, *COVID-19* coronavirus disease 2019, *SD* standard deviation

Note the number of participants included from repeat imaging visit vary slightly based on completeness of cardiac magnetic resonance imaging available

Data are presented as numbers (%), means +/- standard deviation, or medians (interquartile range) as indicated for each demographic variable.

Compared to the controls, cases had, on average, greater deprivation, higher BMI, and higher rates of smoking 37/1054 (3.6%) vs 28/989 (2.9%), and hypertension 238/1054 (23.1%) vs 203/989 (21.2%). Approximately 201/1054 (19%, 176/989 (17.8%) of cases and 17% of controls reported using cardiovascular medications at baseline. The most commonly used medications were statins (14.1% vs 12%) and betablockers (3.4% both cohorts); lower rates of antiplatelet, anticoagulant, and angiotensin receptor blocker use were reported.

Pre-existing cardiovascular disease was rare. The most common conditions were myocardial infarction (2.0% vs 1.8%) and valvular heart disease (1.3% vs 1.0%); heart failure and non-ischemic cardiomyopathies occurred in fewer than 0.5% of participants across both cohorts.

### 3.2 Baseline and repeat ECG and CMR metrics

CMR metrics at baseline and repeat visits are summarized in Table 2. LV and right ventricular (RV) metrics were broadly comparable across the case and control cohorts at both baseline and repeat imaging timepoints. For most metrics, there was no statistically significant difference at either time point.

**Table 2:** Differences in CMR metrics between cases and controls analyzed at baseline and repeat imaging.

Clinical metric name	Baseline imaging			Repeat imaging		
	Control	Cases	P-value	Control	Cases	P-value
*Left ventricle structure, function, and myocardium measurements*						
LV end diastolic volume (mL)	146.0± 31.1	147.3±32.2	0.34	144.7± 31.2	145.1± 31.2	0.75
LV end systolic volume (mL)	88.7±18.2	89.0±19.0	0.72	87.5±17.9	87.2±17.9	0.68
LV ejection fraction (%)	61.2±5.5	60.7±5.7	0.06	61.0±5.7	60.4±6.1	0.02*
LV mass (g)	88.6±22.0	90.1±22.9	0.14	88.8±22.1	90.1±22.4	0.19
LV global longitudinal strain (%)	−18.3±2.1	−18.1±2.2	0.04*	−18.0±2.2	−17.8±2.3	0.03*
LV global circumferential strain (%)	−18.7±2.1	−18.4±2.2	0.001*	−18.6±2.2	−18.3±2.3	0.01*
LV global radial strain (%)	31.2±5.7	30.4±5.7	0.002*	30.8±5.9	30.2±5.8	0.02*
Native T1 (ms)	930.0±38.1	926.9±42.9	0.10	927.1±36.8	930.3±38.3	0.06
*RV structure and function*						
RV end diastolic volume (mL)	151.8±34.9	152.8±36.2	0.48	150.5±34.5	151.7±35.0	0.43
RV end systolic volume (mL)	92.0±20.2	92.3±21.2	0.73	90.7±20.2	90.9±20.3	0.82
RV ejection fraction (%)	61.0±5.8	60.7±5.7	0.35	60.7±5.6	60.4±5.7	0.23
RV global longitudinal strain (%)	−25.1±3.4	−25.1±3.3	0.88	−25.2±3.4	−25.0±3.4	0.37
RV global circumferential strain (%)	−15.2±3.0	−15.1±3.0	0.44	−15.3±3.0	−15.1±3.1	0.23
RV global radial strain (%)	59.0± 14.993	58.9±14.6	0.79	58.8±14.7	58.3±14.8	0.47
*Atrial volumes*						
LA maximum volume (mL)	76.7±24.7	76.5±25.0	0.81	76.6±24.3	76.3±24.7	0.72
LA emptying fraction (%)	65.7±8.8	65.7±9.0	0.97	64.2±8.4	64.1±8.5	0.75
RA maximum volume (mL)	84.6±25.9	83.4±26.2	0.30	84.7±26.1	84.3±27.0	0.71
RA emptying fraction (%)	51.2±8.8	51.6±9.3	0.34	50.7±9.0	50.5±9.1	0.63
*Vascular metrics*						
Ascending aortic strain	0.10±0.06	0.10±0.06	0.46	0.09±0.05	0.09±0.05	0.58
Ascending aorta distensibility (×10^−3^ mmHg^−1^)	1.9±1.4	1.9±1.8	0.62	- 1.66± 1.4	- 1.70±1.5	0.42
Descending aortic strain	0.16±0.05	0.16±0.05	0.41	0.14±0.05	0.14±0.05	0.18
Descending aorta distensibility (×10^−3^ mmHg^−1^)	2.8±1.6	2.8±1.9	0.72	2.6±1.4	2.6±1.7	0.90
*Aortic valve volumes*						
Aortic forward flow volume (mL)	54.8±35.1	54.4±34.3	0.82	58.6±30.9	58.7±30.6	0.96
Aortic backward volume (mL)	−27.0±37.5	−26.1±37.1	0.59	−5.1±3.5	−5.2±3.4	0.81
Aortic mean peak gradient (mmHg)	1.2±0.4	1.2±0.4	0.81	1.1±0.3	1.1±0.441	0.97

*CMR* cardiovascular magnetic resonance, *LV* left ventricle, *RV* right ventricle, *LA* left atrium, *RA* right atrium

The mean value for each metric and standard deviation are shown. The remaining CMR metrics which were assessed but were not clinically relevant or statistically significant can be seen in Supplementary Table 8

* Significant P values from t-tests

Compared to cases, controls had marginally worse global longitudinal strain (GLS), global circumferential strain (GCS), and global radial strain (GRS) at both baseline and repeat imaging visits. There was no difference in baseline LV ejection fraction (LVEF) between cases and controls. At repeat imaging, LVEF was slightly lower in cases compared to controls. While statistically significant, the magnitude of these differences was not clinically relevant (Table 2).

There were no significant differences in myocardial native T1, RV functional metrics, atrial phenotypes, arterial stiffness indicators, aortic flow measures, or any other CMR metrics (Table 2, Supplementary Table 8). In the subset of cases with record of COVID-19 hospitalization, we found no significant differences in the average CMR metrics between cases and controls at either time point (Supplementary Table 9).

There were no significant differences in baseline ECG metrics of cases and controls. At the repeat imaging visit, cases had significantly faster ventricular rate (60 ± 10 bpm vs 59 ± 9 bpm; P = 0.03) and longer corrected QT interval (QTc) (423 ± 24 ms vs 419 ± 23 ms) than controls (Supplementary Table 4). The results for lead–specific ECG biomarkers showed significant differences in QRS interval, R wave duration, S wave area, T wave area, and T wave amplitude (Supplementary Table 10).

### 3.3 Association of baseline CMR and ECG metrics with incident SARS-COV-2

**Fig. 3:**
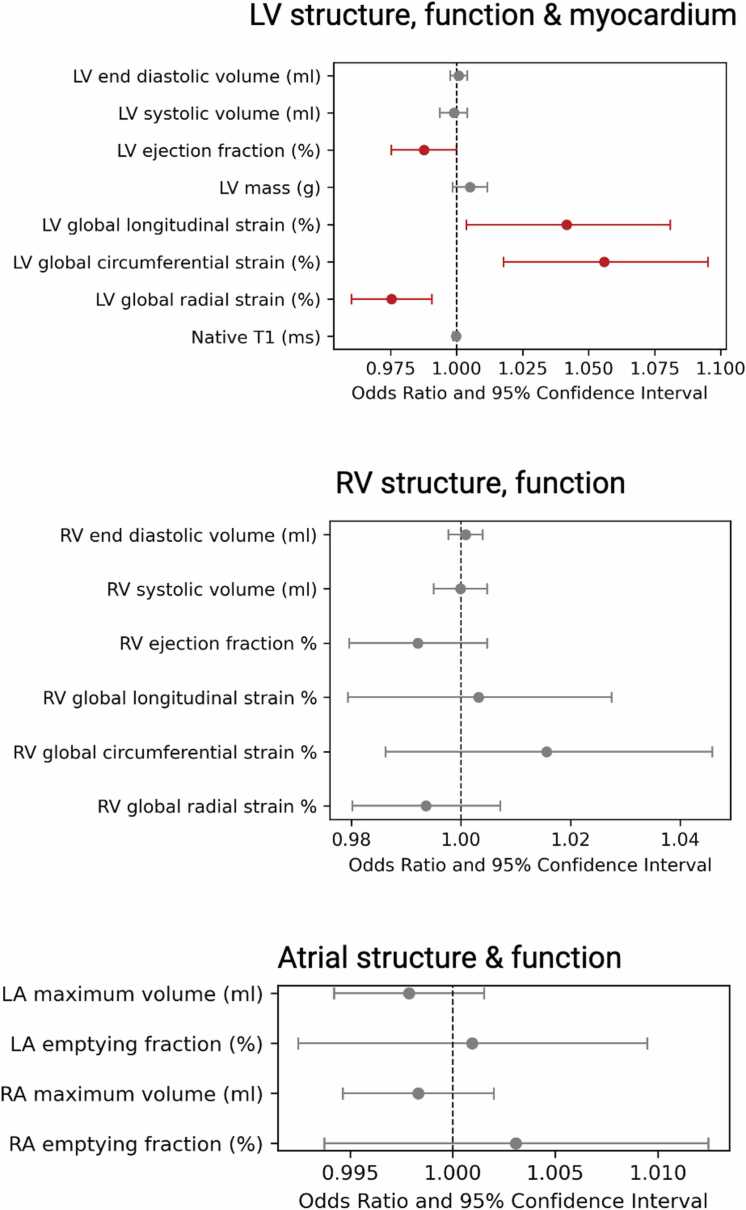
Association between CMR metrics and incident COVID-19. Each line represents results from a separate logistic regression model where the outcome to predict is COVID-19 infection (positive or negative). The models were adjusted for age, sex, ethnicity, deprivation, BMI, smoking, diabetes, hypertension, hypercholesterolemia, and prevalent myocardial infarction. Bars represent the odds ratio and 95% confidence interval per unit increase in the CMR metric. The specific beta coefficient values and P values are shown in Supplementary Table 13. Those highlighted in red are statistically significant results. The results for the remaining CMR metrics that were also analyzed are shown in Supplementary Table 8. *CMR* cardiovascular magnetic resonance, *COVID-19* coronavirus disease 2019, *BMI* body mass index, *LV* left ventricle, *RV* right ventricle, *LA* left atrium, *RA* right atrium. Created using Biorendr.com

In fully adjusted logistic regression models, lower baseline LVEF and poorer LV strain metrics (smaller magnitude of GLS and GCS, i.e., less negative values, and smaller amplitude of GRS, i.e., less positive values) were associated with a higher risk of incident SARS-COV-2 infection (Fig. 3, Supplementary Table 11). Associations between other baseline CMR metrics and infection status were statistically non-significant (Supplementary Table 12).

**Fig. 4:**
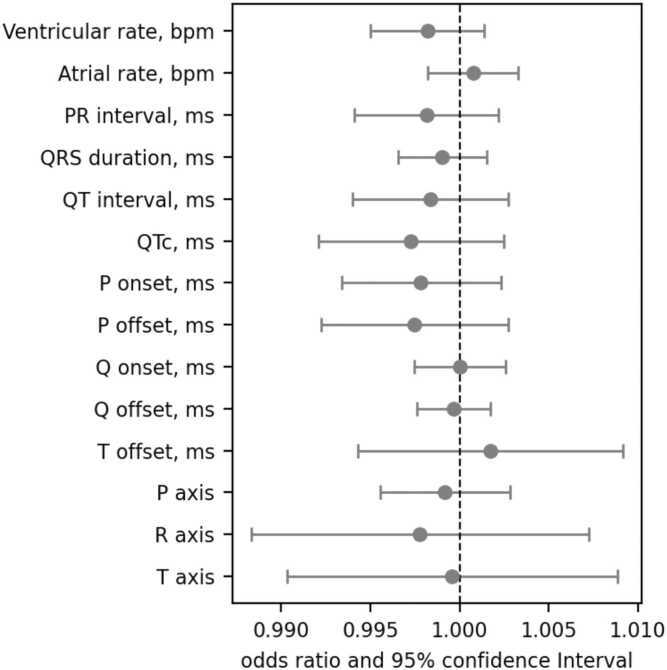
Association between global ECG biomarkers and incident COVID-19 infection. Each line represents results from a separate logistic regression model where the outcome to predict is COVID-19 infection (positive or negative). The models were adjusted for age, sex, ethnicity, deprivation, BMI, smoking, diabetes, hypertension, hypercholesterolemia, and prevalent myocardial infarction. Bars represent the odds ratio and 95% confidence interval per unit increase in ECG markers. The results for the analyzed lead–specific ECG biomarkers are shown in Supplementary Table 13. *ECG* electrocardiographic, *COVID-19* coronavirus disease 2019, *BMI* body mass index, *QTc* corrected QT interval. Created using Biorendr.com

There were no significant associations between baseline global ECG biomarkers and incident SARS-COV-2 infection (Fig. 4). The results for lead–specific ECG biomarkers showed a positive association between T wave area in lead V4 and incident infection (Supplementary Table 13).

### 3.4 Interval change in CMR and ECG metrics and SARS-COV-2

**Fig. 5:**
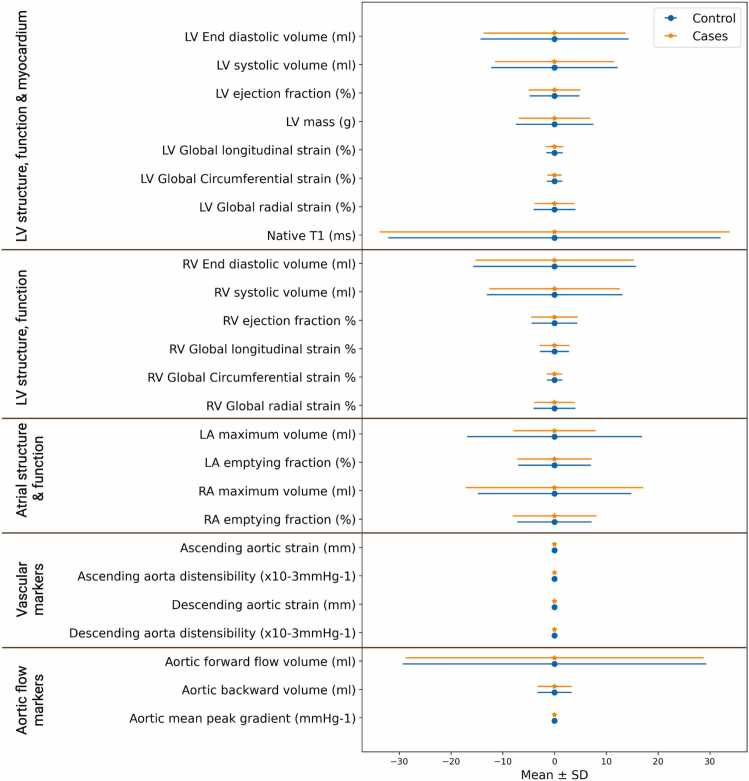
Interval change (difference in predicted and actual values) in CMR metrics between control and cases (infected with COVID-19). Each bar represents the point average residuals and associated standard deviation for each CMR metric. The results for the control group are shown in blue, and the results for the cases are in red. There were no statistically significant results to highlight. *CMR* cardiovascular magnetic resonance, *COVID-19* coronavirus disease 2019, *LV* left ventricle, *RV* right ventricle, *LA* left atrium, *RA* right atrium. Created using Biorendr.com

In analyses considering the degree of change in CMR and ECG metrics at the pre- and post-pandemic timepoints, we found no significant difference in the magnitude of interval change in any of the CMR phenotypes (Table 3, Fig. 5, Supplementary Table 14) or ECG measurements (Supplementary Table 15) between cases and matched uninfected controls.

**Table 3:** Interval change (difference in predicted and actual values) in CMR metrics from baseline to repeat imaging for control and case groups.

Clinical metric name		Control	Cases	P-value
*LV structure, function, and myocardium measurements*				
LV end diastolic volume (mL)		−6.43E−15 ±14.3	1.19E−14± 13.775	0.76
LV end systolic volume (mL)		−6.14E−15±12.2	−8.22E−15±11.551	0.92
LV ejection fraction (%)		−1.11E−14± 4.8	3.54E−15±5.01	0.89
LV mass (g)		−1.9E−14±7.5	−7.07E−15±6.9	0.89
LV global longitudinal strain (%)		−1.59E−16±1.6	2.73E−15±1.7	0.93
LV global circumferential strain (%)		−7.96E−16±1.5	−2.26E−16±1.4	0.96
LV global radial strain (%)		−1.46E−15±4.04	−3.05E−16±3.8	0.92
Native T1 (ms)		1.51E−13±32.1	−7.26E−14±33.8	0.82
*RV structure and function*				
RV end diastolic volume (mL)		2.89E−15±15.7	−9.20E−15±15.3	0.51
RV end systolic volume (mL)		7.48E−15±13.1	−2.06E−15±12.6	0.39
RV ejection fraction (%)		−2.24E−16±4.4	4.32E−15±4.5	0.91
RV global longitudinal strain (%)		−3.06E−15±2.8	7.46E−16±2.8	0.93
RV global circumferential strain (%)		−7.96E−16±1.5	−2.26E−16±1.4	0.96
RV global radial strain (%)		−1.46E−15±4.04	−3.05E−16±3.889	0.92
*Atrial volumes*				
LA maximum volume (mL)		−4.72E−15±16.9	−2.03E−15±17.994	0.83
LA emptying fraction (%)		1.22E−15±7.01	−1.08E−14±7.2	0.93
RA maximum volume (mL)		−1.08E−14±14.8	4.18E−15±17.1	0.75
RA emptying fraction (%)		7.78E−15±7.2	−3.44E−15±8.110	0.56
*Vascular metrics*				
Ascending aortic strain		1.13E−17±0.02	−5.13E−18±0.03	0.67
Ascending aorta distensibility (×10^−3^ mmHg^−1^)		9.74E−17±0.01	2.67E−17±0.01	0.91
Descending aortic strain		3.65E−18±0.02	5.38E−18±0.03	0.67
Descending aorta distensibility (×10^−3^ mmHg^−1^)		−1.59E−16±0.01	5.70E−16±0.01	0.50
*Aortic valve volumes*				
Aortic forward flow volume (mL)		2.07E−15±29.3	5.26E−15±28.8	0.50
Aortic backward volume (mL)		8.21E−17±3.3	3.76E−16±3.331	0.74
Aortic mean peak gradient (mmHg)		−1.81E−16±0.3	−7.69E−17±0.334	0.75

*CMR* cardiovascular magnetic resonance, *LV* left ventricle, *RV* right ventricle, *LA* left atrium, *RA* right atrium

Interval change represents the difference between regressed values for the repeat visit from the baseline visit and the actual values at repeat visit, hence known as standardized residuals (standardized change scores). This value represents the degree of change in the measures from the expected value based on the baseline imaging visit

Fig. 5 helps visualize the results shown in this table

## 4 Discussion

In this cohort of UK Biobank participants with standardized CMR available before and after SARS-COV-2 infection (average 3-year interval), no statistically significant differences were found in the degree of interval change across an extensive range of ECG and CMR phenotypes compared to matched uninfected controls.

The present analysis does not support the association of mild SARS-COV-2 infection with de novo changes in CMR measures of cardiac structure, function, and myocardial tissue character. Analysis of a detailed range of artificial intelligence–derived global and lead–specific ECG metrics revealed no difference in the degree of interval change in pre- and post-infection assessments of cases and controls.

We found an association of poorer baseline (pre-infection) LV function, characterized by LV strain and ejection fraction metrics, with a significantly higher risk of incident SARS-COV-2 infection. These findings are aligned with a previous analysis of the UK Biobank from our group, extending this study to a larger sample and a more comprehensive set of CMR metrics [23]. CMR–derived myocardial strain using feature tracking used in our study is considered a sensitive marker for LV dysfunction [24], [25], [26], [27]. The association of these metrics with incident SARS-COV-2 infection reflects poorer cardiovascular health in cases compared to controls, which is not captured in adjustments for measured traditional cardiovascular risk factors and clinically diagnosed cardiovascular conditions. This observation demonstrates the high potential for reverse causation in cohorts where imaging is unavailable before and after infection for the same individuals.

While cases had sub-clinically poorer baseline LV function metrics, there was no evidence from our analysis that exposure to SARS-COV-2 infection altered the interval change (i.e., the expected change from baseline imaging) in these or any other CMR metric considered. Studies with retrospective [12] and prospective [28] cohorts have reported high rates of CMR abnormalities after SARS-COV-2 infection, even in individuals in whom the acute infection was mild [29]. Skewed patient selection to include symptomatic patients, and timing of CMR scans could explain these results. Other studies have demonstrated that while there is high burden of CMR abnormalities in patients following severe SARS-COV-2 infection requiring hospitalization, this is not significantly greater than in carefully matched comparators [30], [31], [32]. Our findings corroborate these latter suggestions and demonstrate that previous studies reporting persistent CMR abnormalities after recovery from COVID-19 are notably influenced by residual confounding and reverse causation due to the absence of baseline pre-infection imaging and insufficient confounder adjustment.

Particular concern had been raised about persistent myocardial involvement after SARS-COV-2 infection based on abnormalities of myocardial native T1 values [33] with some studies suggesting that this damage can be seen even in young low-risk patients [34] and those with mild infection that did not require hospitalization [28]. Our analysis in a much larger sample of middle-aged people with predominantly asymptomatic or milder community–treated SARS-COV-2 infection, with pre- and post-infection imaging, demonstrates no evidence of global myocardial native T1 abnormalities related to SARS-COV-2 infection. Follow-up studies investigating long COVID have shown that although symptoms may be ongoing, these do not seem to correlate with ongoing CMR abnormalities [35], [36].

Comparing the ECG biomarkers, we found no clinically significant difference in ECG measurements between cases and control groups at both visits, and no association with incident SARS-COV-2 infection. Our study is the largest prospective study to date exploring the ECG manifestations of COVID-19 infection in a community-based population. Our study focused on the interval-based ECG indices guided by current knowledge and evidence in the field. Thakore and colleagues found that QRS and QTc intervals are early markers for COVID-19 disease progression and mortality [37]. In their retrospective study of 828 patients with COVID-19, the majority required hospitalization and 88 intensive care admissions, therefore challenging to illicit the electrophysiological effects due specifically to SARS-COV-2 from those associated with other clinical manifestations. Other studies have also concluded that although there are no strong associations with ECG measurements and COVID-19, the presence of ECG changes increases the odds of death in individuals with the virus. Therefore, while the ECG may be useful for risk stratification in the setting of severe COVID-19, our findings do not suggest a causal association between SARS-COV-2 infection and the occurrence of persistent ECG abnormalities following mild community infection.

## 5 Limitations

The UK Biobank COVID-19 Repeat Imaging Study provided a unique opportunity to assess interval change in ECG and CMR metrics before and after SARS-COV-2 infection, mitigating issues around reverse causation and confounding that had seriously hampered causal inference from previous studies. Our analysis of this dataset provided a granular quality–controlled assessment of volumetric, myocardial strain, aortic flow, myocardial native T1 measurements, and 12-lead ECGs. A key limitation of this study is inherent to the UK Biobank CMR protocol, which has limited non-parametric tissue characterization sequences and does not include contrast-enhanced images. The protocol design was guided by the original remit of the UK Biobank for population studies using imaging techniques with short protocols and minimal risk to participants. The second key limitation is the possibility that those in the control group may have had asymptomatic or possibly very mild undiagnosed SARS-COV-2 infection. In addition, statistical outliers and non-sensical data being excluded in this sample may have contributed to selection bias which would otherwise have been included if manually contoured by an expert. We have reduced the risk of this confounding the results as much as possible with the wide variety of sources (General Practioner, hospitalization, public health laboratories) of infection status. The interval time period (median of 1.3 years) between infection and repeat CMR scan is potentially another limitation, as current literature suggests that any cardiac changes, which are most commonly due to myocarditis [38], may have resolved by the time of the repeat scan. However, the aim of this study is to investigate persistent changes that could be associated with ongoing symptoms. Therefore, the longer interval is appropriate for this study. Another limitation of this study is that most (97%) participants had either mild or asymptomatic SARS-COV-2 infection. The observations in our analysis may not be generalizable to individuals with more severe COVID-19. Our findings do not explain the persistence of symptoms potentially attributable to cardiac dysfunction in people recovered from COVID-19 [39], [40].

## 6 Conclusion

Pre-existing indicators of subclinical LV dysfunction are associated with increased risk of incident SARS-COV-2 infection. There was no evidence to suggest de novo cardiovascular abnormalities or alteration in degree of interval change associated with SARS-COV-2 exposure, across an extensive range of ECG- and CMR-derived metrics. These findings highlight methodological sources of bias in the existing literature and provide reassurance regarding long-term cardiovascular involvement of SARS-COV-2 infection in individuals with mild infection.

## Funding

This work was directly supported by a 10.13039/501100000274British Heart Foundation (BHF) project grant (PG/21/10619). S.C. was funded by the European Union's Horizon 2020 research and innovation program under grant agreement no. 825903 (euCanSHare project). A.S. was supported by a BHF project grant (PG/21/10619). H.N. was supported by the BHF Pat Merriman Clinical Research Training Fellowship (FS/20/22/34640). E.R. is supported by the mini-Centre for Doctoral Training (CDT) award through the Faculty of Science and Engineering, Queen Mary University of London, United Kingdom. Z.R.-E. recognizes the National Institute for Health and Care Research (NIHR) Integrated Academic Training program which supports her Academic Clinical Lectureship post. L.S. was supported by the 10.13039/100015652Barts Charity (G-002389). S.N. and B.R. were supported by the Oxford NIHR Biomedical Research Centre and S.N. by Oxford NIHR Biomedical Research Centre and the Oxford BHF Centre of Research Excellence. S.E.P. acknowledges support from the “SmartHeart” EPSRC program grant (www.nihr.ac.uk; EP/P001009/1) and the European Union's Horizon 2020 research and innovation program under grant agreement No. 825903 (euCanSHare project). This work acknowledges the support of the National Institute for Health and Care Research Barts Biomedical Research Centre (NIHR203330); a delivery partnership of Barts Health NHS Trust, Queen Mary University of London, St George’s University Hospitals NHS Foundation Trust, and St George’s University of London. N.A. recognizes the NIHR Integrated Academic Training program, which supports his Academic Clinical Lectureship post, and acknowledges the support from an 10.13039/501100000691Academy of Medical Sciences Starter Grant for Clinical Lecturers (SGL024\1024). N.C.H. is supported by the UK Medical Research Council (MRC) [MC_PC_21003; MC_PC_21001], and NIHR Southampton Biomedical Research Centre, 10.13039/501100000739University of Southampton, and 10.13039/100010417University Hospital Southampton NHS Foundation Trust, UK. Barts Charity (G-002346) contributed to fees required to access UK Biobank data [access application #2964]. This article is supported by the London Medical Imaging and Artificial Intelligence Centre for Value Based Healthcare (AI4VBH), which is funded from the Data to Early Diagnosis and Precision Medicine strand of the government’s Industrial Strategy Challenge Fund, managed and delivered by Innovate UK on behalf of UK Research and Innovation (UKRI). Views expressed are those of the authors and not necessarily those of the AI4VBH Consortium members, the NHS, Innovate UK, or UKRI. P.M.M. acknowledges generous personal and research support from the Edmond J Safra Foundation and Lily Safra, a NIHR Senior Investigator Award, the UK Dementia Research Institute, the NIHR Biomedical Research Centre and the BHF Centre of Excellence at Imperial College London. The funders did not have any role in the study design, data collection and analysis, decision to publish, or preparation of the manuscript.

## Author contributions

**Aaron Mark Lee:** Writing – original draft, Methodology, Formal analysis. **Alborz Amir-Khalili:** Writing – original draft, Methodology, Formal analysis. **Paul M. Matthews:** Writing – original draft, Methodology. **Wenjia Bai:** Writing – original draft, Methodology. **Sucharitha Chadalavada:** Writing – review & editing, Writing – original draft, Visualization, Supervision, Software, Resources, Methodology, Investigation, Formal analysis, Data curation. **Luca Biasiolli:** Writing – original draft, Investigation. **John P. Greenwood:** Writing – original draft, Methodology, Formal analysis. **Nicholas C. Harvey:** Writing – original draft, Methodology. **Elisa Rauseo:** Writing – review & editing, Writing – original draft, Methodology, Formal analysis, Data curation. **Zahra Raisi-Estabragh:** Writing – review & editing, Writing – original draft, Visualization, Supervision, Methodology, Investigation, Funding acquisition, Formal analysis, Data curation, Conceptualization. **Jackie Cooper:** Writing – review & editing, Writing – original draft, Methodology, Investigation, Formal analysis, Conceptualization. **Stefan Neubauer:** Writing – original draft, Methodology. **Ahmed Salih:** Writing – review & editing, Writing – original draft, Visualization, Methodology, Investigation, Formal analysis, Data curation. **Nay Aung:** Writing – review & editing, Writing – original draft, Software, Methodology, Investigation, Formal analysis. **Hafiz Naderi:** Writing – review & editing, Writing – original draft, Methodology, Formal analysis, Data curation. **Stefan van Duijvenboden:** Writing – review & editing, Writing – original draft, Methodology, Formal analysis. **Anwar A. Chahal:** Writing – review & editing, Writing – original draft, Investigation, Formal analysis. **Gaith S. Dabbagh:** Writing – review & editing, Writing – original draft, Formal analysis. **Steffen E. Petersen:** Writing – review & editing, Writing – original draft, Supervision, Resources, Project administration, Methodology, Investigation, Funding acquisition, Formal analysis, Conceptualization. **Jose D. Vargas:** Writing – review & editing, Writing – original draft, Investigation. **Mihir Sanghvi:** Writing – review & editing, Writing – original draft, Methodology. **Liliana Szabo:** Writing – review & editing, Writing – original draft. **Mohammed Y. Khanji:** Writing – review & editing, Writing – original draft. **Stefan K. Piechnik:** Writing – review & editing, Writing – original draft, Methodology. **Betty Raman:** Writing – review & editing, Writing – original draft, Methodology. **Kenneth Fung:** Writing – review & editing, Writing – original draft, Methodology, Formal analysis. **Jose Paiva:** Writing – review & editing, Writing – original draft, Formal analysis. **Patricia B. Munroe:** Writing – review & editing, Writing – original draft.

## Ethics approval and consent

This study complies with the Declaration of Helsinki; the work was covered by the ethical approval for UK Biobank studies from the NHS National Research Ethics Service on June 17, 2011 (Ref 11/NW/0382) and extended on June 18, 2021 (Ref 21/NW/0157) with written informed consent obtained from all participants.

## Declaration of competing interest

S.E.P. provides consultancy to Cardiovascular Imaging Inc, Calgary, Alberta, Canada. The remaining authors have nothing to disclose. P.M.M. is Chair of the Neurosciences Board of the UKRI MRC, a funder of this study. S.K.P. has patent authorship rights for US patent US20120078084A1.

## Data Availability

This research was conducted using the UK Biobank resource under access application 2964. UK Biobank will make the data available to all bona fide researchers for all types of health-related research in the public interest, without preferential or exclusive access for any persons. All researchers will be subject to the same application process and approval criteria as specified by UK Biobank. For more details on the access procedure, see the UK Biobank website: http://www.ukbiobank.ac.uk/register-apply. This work uses data provided by patients and collected by the NHS as part of their care and support. This research used data assets made available by National Safe Haven as part of the Data and Connectivity National Core Study, led by Health Data Research UK in partnership with the Office for National Statistics and funded by UK Research and Innovation (research which commenced between October 1, 2020 and March 31, 2021 grant ref MC_PC_20029; April 1, 2021 and September 30, 2022 grant ref MC_PC_20058).
