# Supplementary material for: Prospective electrocardiographic and cardiovascular magnetic resonance alterations in the UK Biobank coronavirus disease 2019 repeat imaging study
Source: J Cardiovasc Magn Reson. 2025 Sep 10;27(2):101957. doi: 10.1016/j.jocmr.2025.101957 (PMC12780293; doi:10.1016/j.jocmr.2025.101957)
Supplement: Supplementary file 1 — Supplementary material [file mmc1.docx]

**Supplementary material**

Supplementary Table 1 - settings used for automated batch image analysis

| Image sequence analysed | Batch processing prototype used | Settings for analysis |
| --- | --- | --- |
| SAX and LAX images for volumetric analysis | Circle Inc. CVI 42 prototype 5.14.1.2875 | - The whole of SAX stack was segmented with automated algorithms. - Closed contours were included for volume calculation. - LAX images were also segmented. - Simplified endocardial contour without papillary muscle detection. LV endocardial and epicardial contours were measured both in SAX and LAX cine images (2-chamber, 3-chamber, and 4 chamber). - Right ventricle (RV) surfaces were traced from the SAX and 4-chamber cine CMR views using the automated segmentation tool. - LA and RA contours were automatically drawn in 4-chamber views. - LA area was also segmented in 2-chamber view. |
| Aortic valve phase contrast image | Circle Inc. CVI 42 prototype 5.14.1.2875 | - Machine learning algorithm was used to segment the cross-sectional image of the aorta and the contour was then propagated throughout the whole cardiac cycle on the inner border of the vessel wall, which was defined as the region of interest (ROI). - The aortic flow was automatically measured within ROI. - Background correction and anti-aliasing background correction were not included in the protocol for aortic flow analysis. |
| ShMOLLi image | Circle Inc. CVI 42 prototype 5.14.1.2875 | - Machine learning algorithm was used to segment the endocardial and epicardial borders of the mid-ventricular SAX TI map image into 6 segments. - 10% offset was applied on both endocardium and epicardium contour. |
| SAX and LAX images for feature tracking 2D strain analysis | Circle Inc. CVI 42 (prototype 5.13.7) | - Contours were first drawn with SAX stack segmented only in LV end diastole and LV end systole. - Long axis images were also segmented as the SAX images were. - Temporal smoothing was applied to cines. - LV diastole and systole were to guide tracking. - LV diastole was set as the reference phase for tracking. - SAX images with open LV contours (missing endocardium or epicardium contours) were excluded from the feature tracking. |
| SAX – Short axis images, LAX – Long axis images, LV – Left Ventricle, RV – Right Ventricle, LA – Left atrium, RA – Right atrium | | |

Supplementary Table 2 - Formulas used to calculate vascular markers – aortic strain and aortic distensibility.

| CMR measurement | Formula |
| --- | --- |
| Aortic strain | (Amax – Amin)/Amin |
| Aortic distensibility | (Amax – Amin)/Amin*CPP |
| Amax – Aorta maximum area derived from Aortic systolic mean area  Amin – Aorta minimal area derived from Aortic diastolic mean area  CPP – central pulse pressure derived from pulse wave analysis at respective imaging visit (UK Biobank feid 12678)  The pipeline used to calculate the aortic areas has inbuilt QC which assigns a probability to correct detection of aortic areas. We limited analysis to measurements with correct detection probability > 0.75. | |

Supplementary Table 3 - The number of values included in the study for each CMR metric after applying quality control.

| Clinical metric name & QC parameters set | Baseline imaging | | Repeat imaging | |
| --- | --- | --- | --- | --- |
| LV structure & function measurements | Covid | Control | Covid | Control |
| LV end systolic volume (ml)  10ml < x >120ml | 1045 | 982 | 1041 | 979 |
| LV end diastolic volume (EDV) (ml)  50ml < x > 250ml | 1045 | 980 | 1043 | 981 |
| LV cardiac output (L/min)  2L < x > 10L | 1040 | 980 | 1048 | 981 |
| LV systolic volume (SV) (ml)  30ml < x > 150ml | 1042 | 976 | 1045 | 980 |
| LV mass in systole (g) | 1049 | 983 | 1049 | 984 |
| LV mass (g) | 1049 | 983 | 1049 | 984 |
| LV ejection fraction (EF) (%)  10% < x > 85% | 1044 | 979 | 1049 | 980 |
| Native T1 (ms)  600ms < x > 1500ms | 943 | 885 | 1012 | 954 |
| Diastolic total peak wall thickness (mm) | 1049 | 983 | 1048 | 984 |
| Total peak wall thickness (mm) | 1048 | 980 | 1049 | 983 |
| LV MAPSE lateral (mm) | 1048 | 986 | 1047 | 983 |
| LV MAPSE septal (mm) | 1050 | 985 | 1047 | 983 |
| LV TAPSE (mm) | 1047 | 984 | 1045 | 983 |
| 2ch- LV long axis strain (%) | 1038 | 978 | 1030 | 977 |
| 2ch – LV long axis difference (mm) | 1038 | 981 | 1031 | 977 |
| 2ch – LVAV junction strain (%) | 1039 | 980 | 1029 | 977 |
| 2ch – LVAV junction difference (mm) | 1038 | 980 | 1030 | 977 |
| 2ch LA long axis strain (%) | 1014 | 957 | 1020 | 946 |
| 2ch LA long axis differenece (cm) | 1033 | 976 | 1028 | 972 |
| 2ch LAAV junction strain (%) | 1012 | 955 | 1006 | 939 |
| 2ch LAAV junction difference (mm) | 1033 | 975 | 1026 | 970 |
| 4ch LV long axis strain (%) | 1046 | 983 | 1042 | 982 |
| 4ch LV long axis difference (mm) | 1048 | 984 | 1042 | 982 |
| 4ch LVAV junction strain (%) | 1045 | 982 | 1041 | 982 |
| 4ch LVAV junction difference (mm) | 1048 | 984 | 1041 | 982 |
| 4ch LA long axis strain (%) | 1005 | 949 | 1009 | 949 |
| 4ch LA long axis difference (mm) | 1027 | 970 | 1038 | 970 |
| 4ch LAAV junction strain (%) | 1005 | 949 | 1008 | 952 |
| 4ch LAAV junction difference (mm) | 1029 | 972 | 1039 | 971 |
| 4ch RA long axis strain (%) | 1036 | 981 | 1038 | 976 |
| 4ch RA long axis difference (mm) | 1041 | 983 | 1039 | 978 |
| 4ch RAAV junction strain (%) | 1037 | 981 | 1033 | 978 |
| 4ch RAAV junction difference (mm) | 1041 | 983 | 1038 | 978 |
| 2ch-4ch average long axis strain (%) | 1037 | 978 | 1028 | 976 |
| 2ch-4ch average long axis difference (mm) | 1038 | 978 | 1032 | 976 |
| 2ch-4ch average LVAV junction strain (%) | 1037 | 978 | 1029 | 975 |
| 2ch-4ch average LVAV junction difference (mm) | 1038 | 978 | 1032 | 976 |
| 2ch-4ch average LAX strain (%) | 996 | 955 | 1013 | 942 |
| 2ch-4ch average LA LAX difference (mm) | 1021 | 968 | 1026 | 969 |
| 2ch-4ch average LAAV junction strain (%) | 992 | 950 | 1007 | 937 |
| 2ch-4ch average LAAV junction difference (mm) | 1019 | 967 | 1026 | 967 |
| 2ch LV MAPSE anterior (mm) | 1041 | 981 | 1030 | 974 |
| 2ch LV MAPSE inferior (mm) | 1040 | 978 | 1029 | 977 |
| LV global longitudinal strain (%) | 861 | 834 | 855 | 827 |
| LV global circumferential strain (%) | 861 | 834 | 855 | 827 |
| LV global radial strain (%) | 861 | 834 | 855 | 827 |
| RV structure & function |  |  |  |  |
| RV end systolic volume (ESV) ml  5ml < x > 140ml | 1048 | 982 | 1048 | 983 |
| RV end diastolic volume (EDV) ml  40ml < x > 280ml | 1048 | 982 | 1048 | 982 |
| RV cardiac ouput (L/min)  1L < x > 10L | 1042 | 978 | 1047 | 982 |
| RV systolic volume (SV) ml  20ml < x >170ml | 1045 | 981 | 1048 | 982 |
| RV ejection fraction (EF) %  30% < x >85% | 1044 | 981 | 1048 | 983 |
| RV mass in diastole (g) | 1049 | 983 | 1049 | 984 |
| RV mass in systole (g) | 1048 | 982 | 1049 | 984 |
| RV global longitudinal strain % | 936 | 904 | 940 | 904 |
| RV global circumferential strain % | 936 | 904 | 940 | 904 |
| RV global radial strain % | 936 | 904 | 940 | 904 |
| Atrial structure and function |  |  |  |  |
| LA emptying fraction (%) | 1042 | 979 | 1037 | 974 |
| LA maximum volume (ml) | 1050 | 986 | 1047 | 980 |
| RA maximum volume (ml) | 1046 | 985 | 1043 | 980 |
| RA emptying fraction (%) | 1037 | 977 | 1030 | 976 |
| RA volume at LVES (ml) | 1045 | 985 | 1043 | 980 |
| Vascular metrics |  |  |  |  |
| Mean systolic ascending aortic area (mm^2^) | 840 | 792 | 830 | 758 |
| Mean diastolic ascending aortic area (mm^2^) | 840 | 792 | 830 | 758 |
| Mean systolic descending aortic area (mm^2^) | 840 | 792 | 830 | 758 |
| Mean diastolic descending area (mm^2^) | 840 | 792 | 830 | 758 |
| Aortic valve volumes |  |  |  |  |
| LV peak ejection rate (ml/s) | 1048 | 982 | 1045 | 982 |
| LV peak filling rate (ml/s) | 1047 | 982 | 1048 | 983 |
| RV peak ejection rate (ml/s) | 1047 | 980 | 1049 | 982 |
| RV peak filling rate (ml/s) | 1045 | 981 | 1049 | 983 |
| Aortic total volume (ml) | 977 | 909 | 844 | 787 |
| Aortic regurgitant fraction (%) | 975 | 899 | 1024 | 960 |
| Aortic net positive volume (ml) | 977 | 909 | 1033 | 972 |
| Aortic net negative volume (ml) | 977 | 909 | 888 | 826 |
| Aortic max pressure gradient (mmHg) | 944 | 882 | 1009 | 953 |
| Aortic max flow (ml/s) | 977 | 909 | 1033 | 973 |
| Aortic min flow (ml/s) | 977 | 909 | 844 | 788 |
| Aortic max velocity (cm/s) | 977 | 909 | 1025 | 963 |
| Aortic min velocity (cm/s) | 977 | 909 | 1004 | 946 |
| Aortic max mean velocity (cm/s) | 977 | 909 | 1033 | 973 |
| Aortic max acceleration (cm/s/s) | 977 | 909 | 1033 | 973 |
| Aortic min acceleration (cm/s/s) | 973 | 904 | 1021 | 958 |
| Velocity encoding | 977 | 909 | 1033 | 973 |
| Aortic mean peak gradient | 959 | 885 | 1015 | 959 |
| Aortic backward volume | 977 | 909 | 844 | 787 |
| Aortic forward flow volume | 977 | 909 | 1033 | 972 |
| Quality control measures applied include statistical outliers’ removal at 3 x interquartile and manual outlier removal applied to some metrics detailed next to relevant metric in first column.  Abbreviations: LV – left ventricle; RV – right ventricle; MAPSE – Mitral annular plane systolic excursion; 2ch – Two chamber; 4ch – Four chamber; TAPSE – Tricuspid annular plane systolic excursion; LVAV junction – Left ventricular atrio-ventricular junction; LAAV – Left arterial atrio-ventricular junction; RVAV – right ventricular atrio-ventricular junction; RAAV – Right arterial atrio-ventricular junction; | | | | |

Supplementary Table 4 - Global ECG biomarkers measured in COVID cases and control cohorts at baseline and repeat imaging visits.

| ECG biomarker | Baseline imaging  (n=1,818) | | | Repeat imaging  (n=2,047) | | |
| --- | --- | --- | --- | --- | --- | --- |
|  | Control  (n=873) | Cases  (n=945) | P-value | Control  (n=987) | Cases  (n=1,060) | P-value |
| Ventricular rate, bpm | 60.2 ±9.8 | 60.5 ±9.9 | 0.79 | 58.9 ±9.2 | 60.0 ±10.0 | 0.03 |
| Atrial rate, bpm | 60.2 ±9.7 | 60.3 ±9.7 | 0.93 | 58.7 ±9.1 | 59.7 ±9.8 | 0.06 |
| PR interval, ms | 163.7 ±25.6 | 163.3 ±25.7 | 0.68 | 166.6 ±27.6 | 165.5 ±26.8 | 0.40 |
| QRS duration, ms | 87.3 ±12.1 | 87.8 ±11.8 | 0.34 | 86.4 ±12.5 | 86.1 ±11.7 | 0.80 |
| QT interval, ms | 420.6 ±31.2 | 421.4 ±31.9 | 0.63 | 427.2 ±30.4 | 425.9 ±31.5 | 0.61 |
| QTc, ms | 417.2 ±22.4 | 418.8 ±23.8 | 0.18 | 419.3 ±22.9 | 421.9 ±23.7 | 0.005 |
| P onset, ms | 137.2 ±13.8 | 137.2 ±13.5 | 0.87 | 135.6 ±14.8 | 136.0 ±14.2 | 0.44 |
| P offset, ms | 186.6 ±14.1 | 186.3 ±14.0 | 0.92 | 183.9 ±15.2 | 184.6 ±14.2 | 0.36 |
| Q onset, ms | 219.1 ±4.7 | 218.8 ±4.7 | 0.31 | 219.0 ±4.9 | 218.8 ±5.1 | 0.51 |
| Q offset, ms | 262.7 ±6.1 | 262.6 ±6.0 | 0.96 | 262.3 ±6.5 | 262.1 ±6.5 | 0.91 |
| T offset, ms | 429.4 ±16.0 | 429.4 ±16.2 | 0.86 | 432.4 ±15.1 | 431.6 ±16.2 | 0.54 |
| P axis, ° | 49.7 ±24.4 | 48.6 ±24.0 | 0.71 | 49.9 ±22.5 | 48.9 ±22.4 | 0.32 |
| R axis, ° | 32.2 ±39.2 | 32.5 ±38.4 | 0.84 | 28.4 ±35.0 | 27.4 ±33.4 | 0.36 |
| T axis, ° | 40.6 ±31.4 | 38.8 ±30.2 | 0.09 | 40.0 ±25.7 | 38.4 ±24.9 | 0.06 |
| ECG – electrocardiogram, bpm - beats per minute, ms - milliseconds | | | | | | |

Supplementary Table 5 - ECG biomarker definitions

| ECG biomarker | Definition |
| --- | --- |
| Ventricular rate | Number of QRS complexes in 60 seconds (bpm) |
| Atrial rate | Number of atrial complexes in 60 seconds (bpm) |
| PR interval | Start of P wave to start of QRS complex (ms) |
| QRS duration | QRS duration (ms) |
| QT interval | Start of Q to the end of the T wave |
| QTc | Corrected QT interval was calculated using the Bazett’s formula. |
| P onset | Start of P wave |
| P offset | End of P wave |
| P peak amplitude | Peak of P wave in reference to the median complex (µV) |
| P duration | Start of P to end of P wave (ms) |
| P area | Area under the P wave (µv/ms) |
| P axis | P wave axis (°) |
| Q onset | Start of Q wave (ms) |
| Q offset | End of Q wave (ms) |
| Q peak amplitude | Peak height of Q wave (µv) |
| Q duration | Start of Q to end of Q wave (ms) |
| Q area | Area under the Q wave (µv/ms) |
| R peak amplitude | Maximal R wave amplitude (µv) |
| R duration | Start of R to end of R wave (ms) |
| R area | Area under the R wave (µv/ms) |
| R axis | R wave axis (°) |
| S peak amplitude | Peak of S wave in reference to the median complex (µV) |
| S duration | Start of S to end of S wave (ms) |
| S area | Area under the S wave (µv/ms) |
| QRS area | Area under the QRS wave (µv/ms) |
| T onset | Start of T wave (ms) |
| T offset | End of T wave (ms) |
| T duration | Start of T to end of T wave (ms) |
| T area | Area under the T wave (µv/ms) |
| T peak amplitude | Peak height of T wave (µv) |
| T axis | T wave axis (°) |

Supplementary Table 6 —ECG biomarkers were chosen for analysis in the study, and quality control (QC) measures were applied (statistical outliers were removed at 3 x interquartile).

| ECG biomarker | Baseline | | Repeat | |
| --- | --- | --- | --- | --- |
|  | Covid | Control | Covid | Control |
| Atrial rate | 942 | 873 | 1057 | 986 |
| P area I | 938 | 867 | 1057 | 987 |
| P area II | 945 | 873 | 1060 | 987 |
| P area V1 | 945 | 873 | 1045 | 987 |
| P area V2 | 941 | 871 | 1060 | 987 |
| P area V3 | 943 | 871 | 1057 | 987 |
| P area V4 | 943 | 873 | 1059 | 987 |
| P area V5 | 944 | 872 | 1058 | 987 |
| P area V6 | 942 | 872 | 1058 | 987 |
| P duration I | 915 | 856 | 1024 | 968 |
| P duration II | 881 | 815 | 1021 | 963 |
| P duration V1 | 945 | 873 | 1060 | 987 |
| P_duration V2 | 945 | 873 | 1060 | 987 |
| P duration V3 | 945 | 873 | 1060 | 987 |
| P duration V4 | 926 | 858 | 1036 | 975 |
| P duration V5 | 924 | 853 | 1032 | 969 |
| P duration V6 | 909 | 841 | 1032 | 965 |
| P amplitude I | 895 | 845 | 1052 | 979 |
| P amplitude II | 942 | 870 | 1055 | 981 |
| P amplitude V1 | 945 | 873 | 1060 | 987 |
| P amplitude V2 | 938 | 870 | 1060 | 985 |
| P amplitude V3 | 935 | 859 | 1047 | 975 |
| P amplitude V4 | 939 | 866 | 1054 | 985 |
| P amplitude V5 | 941 | 865 | 1053 | 984 |
| P amplitude V6 | 940 | 870 | 1051 | 984 |
| PR interval | 941 | 873 | 1056 | 987 |
| P axis | 944 | 872 | 1059 | 986 |
| P offset | 943 | 872 | 1056 | 986 |
| P onset | 943 | 873 | 1057 | 986 |
| Q area_I | 926 | 851 | 1048 | 978 |
| Q area II | 929 | 853 | 1036 | 963 |
| Q area V1 | 879 | 803 | 973 | 908 |
| Q area V2 | 913 | 824 | 971 | 900 |
| Q area V3 | 908 | 829 | 1009 | 930 |
| Q area V4 | 785 | 704 | 850 | 780 |
| Q area V5 | 907 | 837 | 1012 | 932 |
| Q area V6 | 928 | 862 | 1035 | 962 |
| Q duration I | 942 | 869 | 1059 | 985 |
| Q duration II | 943 | 871 | 1055 | 986 |
| Q duration V1 | 879 | 803 | 973 | 908 |
| Q duration V2 | 913 | 824 | 971 | 900 |
| Q duration V3 | 908 | 829 | 1009 | 930 |
| Q duration V4 | 785 | 704 | 850 | 780 |
| Q duration V5 | 943 | 870 | 1058 | 987 |
| Q duration V6 | 944 | 872 | 1057 | 987 |
| Q amplitude I | 939 | 862 | 1056 | 985 |
| Q amplitude II | 937 | 864 | 1050 | 982 |
| Q amplitude V1 | 879 | 803 | 973 | 908 |
| Q amplitude V2 | 913 | 824 | 971 | 900 |
| Q amplitude V3 | 908 | 829 | 1009 | 930 |
| Q amplitude V4 | 785 | 704 | 850 | 780 |
| Q amplitude V5 | 926 | 856 | 1046 | 964 |
| Q amplitude V6 | 940 | 868 | 1050 | 977 |
| QT interval | 944 | 873 | 1058 | 987 |
| Q offset | 935 | 867 | 1053 | 979 |
| Q onset | 940 | 872 | 1057 | 982 |
| QRS area I | 937 | 870 | 1053 | 982 |
| QRS area II | 942 | 871 | 1056 | 984 |
| QRS area V1 | 932 | 860 | 1044 | 957 |
| QRS area V2 | 940 | 869 | 1053 | 976 |
| QRS area V3 | 939 | 870 | 1051 | 980 |
| QRS area V4 | 940 | 870 | 1056 | 985 |
| QRS area V5 | 940 | 873 | 1057 | 984 |
| QRS area V6 | 939 | 871 | 1054 | 983 |
| QRS duration | 931 | 868 | 1038 | 972 |
| QRS interval I | 933 | 861 | 1046 | 968 |
| QRS interval II | 933 | 848 | 1041 | 978 |
| QRS interval V1 | 929 | 855 | 1035 | 961 |
| QRS interval V2 | 939 | 869 | 1049 | 980 |
| QRS interval V3 | 944 | 873 | 1057 | 987 |
| QRS interval V4 | 942 | 872 | 1052 | 982 |
| QRS interval V5 | 936 | 867 | 1042 | 977 |
| QRS interval V6 | 933 | 862 | 1045 | 967 |
| cQT calculation | 942 | 873 | 1057 | 987 |
| R area I | 940 | 871 | 1052 | 981 |
| R area II | 944 | 871 | 1059 | 986 |
| R area V1 | 923 | 854 | 1045 | 974 |
| R area V2 | 931 | 861 | 1047 | 967 |
| R area V3 | 942 | 870 | 1052 | 985 |
| R area V4 | 943 | 872 | 1056 | 984 |
| R area V5 | 942 | 873 | 1057 | 985 |
| R area V6 | 940 | 871 | 1055 | 984 |
| R duration I | 944 | 873 | 1059 | 986 |
| R duration II | 945 | 873 | 1059 | 987 |
| R duration V1 | 933 | 861 | 1048 | 975 |
| R duration V2 | 934 | 865 | 1047 | 967 |
| R duration V3 | 935 | 867 | 1050 | 971 |
| R duration V4 | 931 | 859 | 1053 | 969 |
| R duration V5 | 939 | 865 | 1053 | 982 |
| R duration V6 | 943 | 871 | 1054 | 983 |
| R amplitude I | 945 | 872 | 1059 | 987 |
| R amplitude II | 943 | 873 | 1059 | 986 |
| R amplitude V1 | 935 | 861 | 1051 | 981 |
| R amplitude V2 | 940 | 870 | 1058 | 982 |
| R amplitude V3 | 944 | 871 | 1055 | 987 |
| R amplitude V4 | 944 | 872 | 1060 | 987 |
| R amplitude V5 | 942 | 872 | 1060 | 985 |
| R amplitude V6 | 942 | 870 | 1058 | 986 |
| R axis | 944 | 872 | 1060 | 987 |
| S area I | 889 | 810 | 1009 | 903 |
| S area II | 910 | 836 | 1009 | 952 |
| S area V1 | 944 | 870 | 1057 | 981 |
| S area V2 | 941 | 869 | 1052 | 981 |
| S area V3 | 939 | 868 | 1051 | 975 |
| S area V4 | 939 | 865 | 1053 | 976 |
| S area V5 | 936 | 862 | 1052 | 971 |
| S area V6 | 919 | 853 | 1024 | 964 |
| S duration I | 945 | 873 | 1060 | 987 |
| S duration II | 945 | 873 | 1058 | 987 |
| S duration V1 | 945 | 873 | 1060 | 986 |
| S duration V2 | 943 | 872 | 1054 | 983 |
| S duration V3 | 941 | 872 | 1052 | 981 |
| S duration V4 | 939 | 870 | 1054 | 984 |
| S duration V5 | 943 | 873 | 1054 | 986 |
| S duration V6 | 945 | 873 | 1060 | 986 |
| S amplitude I | 926 | 848 | 1043 | 964 |
| S amplitude II | 933 | 863 | 1049 | 979 |
| S amplitude V1 | 944 | 871 | 1057 | 985 |
| S amplitude V2 | 943 | 871 | 1059 | 986 |
| S amplitude V3 | 941 | 871 | 1057 | 985 |
| S amplitude V4 | 944 | 871 | 1059 | 985 |
| S amplitude V5 | 942 | 868 | 1057 | 978 |
| S amplitude V6 | 939 | 867 | 1033 | 977 |
| T area I | 938 | 871 | 1059 | 985 |
| T area II | 942 | 873 | 1059 | 986 |
| T area V1 | 944 | 872 | 1059 | 986 |
| T area V2 | 945 | 872 | 1060 | 986 |
| T area V3 | 942 | 870 | 1058 | 984 |
| T area V4 | 938 | 872 | 1056 | 986 |
| T area V5 | 937 | 872 | 1059 | 985 |
| T area V6 | 937 | 873 | 1055 | 985 |
| T duration I | 908 | 847 | 1020 | 954 |
| T duration II | 867 | 802 | 1001 | 946 |
| T duration V1 | 945 | 873 | 1060 | 987 |
| T duration V2 | 925 | 859 | 1027 | 961 |
| T duration V3 | 927 | 864 | 1038 | 966 |
| T duration V4 | 929 | 861 | 1035 | 969 |
| T duration V5 | 925 | 852 | 1032 | 974 |
| T duration V6 | 920 | 843 | 1027 | 968 |
| T end I | 939 | 863 | 1046 | 984 |
| T end II | 943 | 871 | 1056 | 982 |
| T end V1 | 944 | 871 | 1057 | 987 |
| T end V2 | 941 | 872 | 1056 | 986 |
| T end V3 | 938 | 869 | 1058 | 982 |
| T end V4 | 943 | 871 | 1058 | 986 |
| T end V5 | 937 | 864 | 1055 | 985 |
| T end V6 | 936 | 865 | 1055 | 984 |
| T amplitude I | 938 | 869 | 1056 | 982 |
| T amplitude II | 941 | 871 | 1059 | 987 |
| T amplitude V1 | 945 | 872 | 1060 | 986 |
| T amplitude V2 | 945 | 872 | 1060 | 987 |
| T amplitude V3 | 943 | 872 | 1059 | 985 |
| T amplitude V4 | 938 | 873 | 1057 | 986 |
| T amplitude V5 | 937 | 872 | 1058 | 984 |
| T amplitude V6 | 939 | 873 | 1056 | 986 |
| T axis | 921 | 853 | 1049 | 983 |
| T offset | 943 | 873 | 1058 | 987 |
| Ventricular rate | 942 | 873 | 1059 | 987 |

Supplementary Table 7 - Defining covariates of interest in UK Biobank

| Covariate | UKBB data field(s) | Instance or code |
| --- | --- | --- |
| Age | 21003 | 2 at imaging |
| Sex | 31 | - |
| BMI | 23104 | 2 at imaging |
| Ethnicity group | 21000 | - |
| Smoking | 20116 (smoking status) | 2, at imaging |
| Alcohol | 20117 (Alcohol drinker status) | 2, at imaging |
| Townsend deprivation | 189 | - |
| Diabetes | Self-report (20002) | 1220, diabetes  1222, type 1 diabetes  1223, type 2 diabetes |
|  | Medication for cholesterol, blood pressure or diabetes (6177) | 3, Insulin |
|  | ICD10 (41202) | E100, Type 1 diabetes mellitus: With coma  E101, Type 1 diabetes mellitus: With ketoacidosis  E102, Type 1 diabetes mellitus: With renal complications  E103, Type 1 diabetes mellitus: With ophthalmic complications  E104, Type 1 diabetes mellitus: With neurological complications  E105, Type 1 diabetes mellitus: With peripheral circulatory complications  E106, Type 1 diabetes mellitus: With other specified complications  E107, Type 1 diabetes mellitus: With multiple complications  E108, Type 1 diabetes mellitus: With unspecified complications  E109, Type 1 diabetes mellitus: Without complications  E110, Type 2 diabetes mellitus: With coma  E111, Type 2 diabetes mellitus: With ketoacidosis  E112, Type 2 diabetes mellitus: With renal complications  E113, Type 2 diabetes mellitus: With ophthalmic complications  E114, Type 2 diabetes mellitus: With neurological complications  E115, Type 2 diabetes mellitus: With peripheral circulatory complications  E116, Type 2 diabetes mellitus: With other specified complications  E117, Type 2 diabetes mellitus: With multiple complications  E118, Type 2 diabetes mellitus: With unspecified complications  E119, Type 2 diabetes mellitus: Without complications  E130, Other specified diabetes mellitus: With coma  E131, Other specified diabetes mellitus: With ketoacidosis  E132, Other specified diabetes mellitus: With renal complications  E133, Other specified diabetes mellitus: With ophthalmic complications  E134, Other specified diabetes mellitus: With neurological complications  E135, Other specified diabetes mellitus: With peripheral circulatory complications  E136, Other specified diabetes mellitus: With other specified complications  E137, Other specified diabetes mellitus: With multiple complications  E138, Other specified diabetes mellitus: With unspecified complications  E139, Other specified diabetes mellitus: Without complications  E140, Unspecified diabetes mellitus: With coma  E141, Unspecified diabetes mellitus: With ketoacidosis  E142, Unspecified diabetes mellitus: With renal complications  E143, Unspecified diabetes mellitus: With ophthalmic complications  E144, Unspecified diabetes mellitus: With neurological complications  E145, Unspecified diabetes mellitus: With peripheral circulatory complications  E146, Unspecified diabetes mellitus: With other specified complications  E147, Unspecified diabetes mellitus: With multiple complications  E148, Unspecified diabetes mellitus: With unspecified complications  E149, Unspecified diabetes mellitus: Without complications  G590, Diabetic mononeuropathy  G632, Diabetic polyneuropathy  H280, Diabetic cataract  H360, Diabetic retinopathy  M142, Diabetic arthropathy  N083, Glomerular disorders in diabetes mellitus  O240, Diabetes mellitus in pregnancy: Pre-existing type 1 diabetes mellitus  O241, Diabetes mellitus in pregnancy: Pre-existing type 2 diabetes mellitus  O243, Diabetes mellitus in pregnancy: Pre-existing diabetes mellitus, unspecified  O244, Diabetes mellitus arising in pregnancy  O249, Diabetes mellitus in pregnancy, unspecified  Y423, Insulin and oral hypoglycaemic [antidiabetic] drugs |
| Type 1 diabetes | Self-report (20002) | 1222, type 1 diabetes |
| Type 1 diabetes | Self-report (20002) | 1223, type 1 diabetes |
| Hypertension | Self-report (20002) | 1065, hypertension  1072, essential hypertension () |
|  | Medication for cholesterol, blood pressure or diabetes (6177) | 2, Blood pressure medication |
|  | ICD10 (41202) | I10X, Essential (primary) hypertension  I110, Hypertensive heart disease with (congestive) heart failure  I119, Hypertensive heart disease without (congestive) heart failure  I120, Hypertensive renal disease with renal failure  I129, Hypertensive renal disease without renal failure  I130, Hypertensive heart and renal disease with (congestive) heart failure  I131, Hypertensive heart and renal disease with renal failure  I132, Hypertensive heart and renal disease with both (congestive) heart failure and renal failure  I139, Hypertensive heart and renal disease, unspecified |
| High cholesterol | Self-report (20002) | 1473, high cholesterol |
|  | Medication for cholesterol, blood pressure or diabetes (6177) | 1, Cholesterol lowering medication |
|  | ICD10 (41202) | E780, Pure hypercholesterolaemia  E782, Mixed hyperlipidaemia  E783, Hyperchylomicronaemia  E784, Other hyperlipidaemia  E785, Hyperlipidaemia, unspecified |
| Prevalent myocardial infarction | Self-report (20002) | 1075, heart attack/myocardial infarction |
| Acute Myocardial Infarction (AMI) | ICD10 (41202) | I210, Acute transmural myocardial infarction of anterior wall  I211, Acute transmural myocardial infarction of inferior wall  I212, Acute transmural myocardial infarction of other sites  I213, Acute transmural myocardial infarction of unspecified site  I214, Acute subendocardial myocardial infarction  I219, Acute myocardial infarction, unspecified |
| Hospitalization for COVID | ICD10 (41202) | U071, COVID-19 virus identified |

Supplementary Table 8 - Differences in CMR metrics analyzed between cases and controls at baseline and repeat imaging.

| Clinical metric name | Baseline imaging | | | Repeat imaging | | |
| --- | --- | --- | --- | --- | --- | --- |
| LV structure & function measurements | Control | Cases | P-value | Control | Cases | P-value |
| LV end systolic volume (ml) | 57.2 ±16.8 | 58.2 ±17.02 | 0.18 | 56.9 ±17.2 | 57.8 ±17.2 | 0.25 |
| LV cardiac output (L/min) | 5.4 ±1.2 | 5.4 ±1.2 | 0.98 | 5.12 ±1.13 | 5.2 ±1.1 | 0.13 |
| LV mass in systole (g) | 87.02 ± 23.1 | 88.5 ± 23.8 | 0.15 | 86.9 ± 22.8 | 88.4 ± 23.1 | 0.14 |
| Diastolic total peak wall thickness (mm) | 9.7 ± 1.6 | 9.8 ± 1.6 | 0.15 | 9.8 ± 1.6 | 9.9 ± 1.61 | 0.12 |
| Total peak wall thickness (mm) | 9.7 ± 1.6 | 9.8 ± 1.6 | 0.15 | 9.8 ± 1.6 | 9.9 ± 1.6 | 0.12 |
| LV MAPSE lateral (mm) | 16.4 ± 3.2 | 16.1 ± 3.1 | 0.07 | 16.0 ± 3.0 | 15.7 ± 3.1 | 0.05 |
| LV MAPSE septal (mm) | 14.1 ± 2.9 | 13.8 ± 3.09 | 0.08 | 13.4 ± 2.9 | 13.4 ± 2.9 | 0.71 |
| LV TAPSE (mm) | 23.3 ± 4.5 | 23.2 ± 4.7 | 0.60 | 22.9 ± 4.6 | 22.9 ± 4.7 | 0.86 |
| 2ch- LV long axis strain (%) | -18.5 ± 3.0 | -18.3 ± 2.9 | 0.13 | -18.1 ± 3.0 | -17.9 ± 3.1 | 0.52 |
| 2ch – LV long axis difference (mm) | -17.5 ± 3.2 | -17.4 ± 3.1 | 0.32 | -17.0 ± 3.1 | -16.9 ± 3.2 | 0.43 |
| 2ch – LVAV junction strain (%) | -17.9 ± 2.9 | -17.7 ± 2.8 | 0.12 | -17.4 ± 2.8 | -17.3 ± 2.9 | 0.51 |
| 2ch – LVAV junction difference (mm) | -17.4 ± 3.1 | -17.3 ± 3.09 | 0.23 | -16.9 ± 3.03 | -16.8 ± 3.1 | 0.34 |
| 2ch LA long axis strain (%) | 52.2 ± 24.6 | 52.7 ± 23.8 | 0.62 | 52.3 ± 25.8 | 51.5 ± 25.7 | 0.44 |
| 2ch LA long axis differenece (cm) | 17.1 ± 5.05 | 17.2 ± 4.9 | 0.47 | 16.8 ± 5.1 | 16.5 ± 4.8 | 0.10 |
| 2ch LAAV junction strain (%) | 43.3 ± 18.7 | 43.5 ± 18.3 | 0.80 | 43.0 ± 19.2 | 41.7 ± 18.5 | 0.13 |
| 2ch LAAV junction difference (mm) | 15.8 ± 4.6 | 15.9 ± 4.6 | 0.56 | 15.6 ± 4.8 | 15.2 ± 4.5 | 0.06 |
| 4ch LV long axis strain (%) | -18.5 ± 3.6 | -18.2 ± 3.6 | 0.11 | -17.9 ± 3.3 | -17.9 ± 3.6 | 0.59 |
| 4ch LV long axis difference (mm) | -17.0 ± 3.5 | -16.7 ± 3.5 | 0.12 | -16.2 ± 3.1 | -16.3.51 | 0.49 |
| 4ch LVAV junction strain (%) | -17.6 ± 3.4 | -17.4 ± 3.4 | 0.08 | -17.1 ± 3.1 | -17.1 ± 3.4 | 0.99 |
| 4ch LVAV junction difference (mm) | -16.7 ± 3.4 | -16.4 ± 3.4 | 0.11 | -16.0 ± 3.1 | -16.1 ± 3.4 | 0.86 |
| 4ch LA long axis strain (%) | 26.6 ± 10.9 | 26.4 ± 10.09 | 0.66 | 25.1 ± 10.08 | 24.9 ± 10.1 | 0.63 |
| 4ch LA long Axis difference (mm) | 12.4 ± 3.8 | 12.4 ± 3.7 | 0.95 | 11.9 ± 3.7 | 12.0 ± 3.8 | 0.81 |
| 4ch LAAV junction strain (%) | 25.0 ± 9.7 | 24.8 ± 9.2 | 0.70 | 23.7 ± 9.3 | 23.3 ± 9.1 | 0.38 |
| 4ch LAAV junction difference (mm) | 12.2 ± 3.7 | 12.2 ± 3.8 | 0.93 | 11.7 ± 3.6 | 11.7 ± 3.8 | 0.82 |
| 4ch RA long axis strain (%) | 36.6 ± 10.9 | 36.3 ± 10.7 | 0.54 | 35.3 ± 11.1 | 35.1 ± 10.9 | 0.60 |
| 4ch RA long axis difference (mm) | 14.9 ± 3.5 | 14.9 ± 3.6 | 0.84 | 14.7 ± 3.6 | 14.6 ± 3.6 | 0.62 |
| 4ch RAAV junction strain (%) | 32.8 ± 9.5 | 32.8 ± 9.6 | 0.90 | 31.7 ± 9.9 | 31.3 ± 9.3 | 0.30 |
| 4ch RAAV junction difference (mm) | 14.3 ± 3.3 | 14.4 ± 3.5 | 0.64 | 14.1 ± 3.4 | 14.0 ± 3.4 | 0.64 |
| 2ch-4ch average long axis strain (%) | -18.5 ± 2.9 | -18.4 ± 2.8 | 0.21 | -17.9 ± 2.7 | -18.0 ± 2.9 | 0.92 |
| 2ch-4ch average long axis difference (mm) | -17.3 ± 2.9 | -17.1 ± 2.9 | 0.25 | -16.6 ± 2.7 | -16.6 ± 2.9 | 0.79 |
| 2ch-4ch average LVAV junction strain (%) | -17.8 ± 2.7 | -17.6 ± 2.7 | 0.14 | -17.2 ± 2.6 | -17.2 ± 2.8 | 0.71 |
| 2ch-4ch average LVAV junction difference (mm) | -17.1 ± 2.8 | -16.9 ± 2.8 | 0.18 | -16.4 ± 2.6 | -16.4 ± 2.9 | 0.96 |
| 2ch-4ch average LAX strain (%) | 40.5 ± 15.9 | 40.4 ± 14.9 | 0.96 | 39.7 ± 16.1 | 39.2 ± 15.7 | 0.48 |
| 2ch-4ch average LA LAX difference (mm) | 14.8 ± 3.6 | 14.9 ± 3.6 | 0.17 | 14.6 ± 3.5 | 14.3 ± 3.5 | 0.10 |
| 2ch-4ch average LAAV junction strain (%) | 34.8 ± 12.4 | 34.8 ± 11.7 | 0.97 | 34.2 ± 12.7 | 33.7 ± 12.6 | 0.40 |
| RV structure & function |  |  |  |  |  |  |
| RV end systolic volume | 59.8 ±18.7 | 60.5 ±18.9 | 0.38 | 59.8 ±18.2 | 60.7 ±18.5 | 0.26 |
| RV cardiac output (L/min) | 5.6 ±1.2 | 5.6 ±1.3 | 0.39 | 5.3 ±1.2 | 5.4 ±1.2 | 0.06 |
| RV mass in diastole (g) | 38.3 ±6.8 | 38.4 ±6.9 | 0.77 | 37.9 ±6.6 | 38.1 ±6.7 | 0.42 |
| RV mass in systole (g) | 21.7 ± 4.7 | 21.9 ± 4.8 | 0.25 | 21.7 ± 4.6 | 21.9 ± 4.7 | 0.21 |
| Vascular metrics |  |  |  |  |  |  |
| Mean systolic ascending aortic area (mm^2^) | 778.5 ±179.3 | 765.5 ±170.7 | 0.13 | 802.5 ±186.2 | 794.3 ±179.9 | 0.37 |
| Mean diastolic ascending aortic area (mm^2^) | 709.1 ±175.7 | 699.1 ±171.2 | 0.24 | 740.0±180 | 732.2 ±177.2 | 0.38 |
| Mean systolic descending aortic area (mm^2^) | 466.9 ±96.1 | 469.1 ±93.9 | 0.62 | 481.2 ±94.8 | 482.0 ±98.0 | 0.86 |
| Mean diastolic descending area (mm^2^) | 405.1 ±90.3 | 408.01 ±89.5 | 0.50 | 421.8 ±89.2 | 423.8 ±92.1 | 0.66 |
| Aortic valve volumes |  |  |  |  |  |  |
| LV peak ejection rate (ml/s) | 640.3 ± 155.3 | 637.1 ± 158.6 | 0.64 | 607.5 ± 145.4 | 614.4 ± 150.1 | 0.29 |
| LV peak filling rate (ml/s) | 559.5 ± 140.6 | 560.3 ± 148.3 | 0.89 | 523.7 ± 134.8 | 531.1 ± 139.4 | 0.22 |
| RV peak ejection rate (ml/s) | 629.9 ± 169.8 | 643.6 ± 176.9 | 0.07 | 609. ± 161.6 | 622.3 ± 171.3 | 0.07 |
| RV peak filling rate (ml/s) | 623.3 ± 170.1 | 628.4 ± 178.4 | 0.50 | 590. ± 165.7 | 599.7 ± 173.9 | 0.23 |
| Aortic total volume (ml) | 27.8 ± 70.5 | 28.3 ± 69.2 | 0.86 | 66.8 ± 15. | 66.2 ± 15.7 | 0.49 |
| Aortic regurgitant fraction (%) | 5.2 ± 3.9 | 5.4.29 | 0.13 | 5.98 ± 4.31 | 6.2 ± 4.4 | 0.31 |
| Aortic net positive volume (ml) | 65.8 ± 39.4 | 64.9 ± 38.5 | 0.66 | 70.9 ± 35.5 | 70.7 ± 35.0 | 0.87 |
| Aortic net negative volume (ml) | -40.7 ± 42.8 | -39.7 ± 42.8 | 0.59 | -20.1 ± 15.7 | -19.9 ± 15.6 | 0.79 |
| Aortic max pressure gradient (mmHg) | 5.9 ± 2.3 | 5.8 ± 2.4 | 0.41 | 5.5 ± 2.2 | 5.4 ± 2.1 | 0.35 |
| Aortic max flow (ml/s) | 276.6 ± 169.1 | 277.2 ± 167.3 | 0.93 | 289.03 ± 146.04 | 291.02 ± 145. | 0.76 |
| Aortic min flow (ml/s) | -129.8 ± 163.8 | -125.8 ± 160.8 | 0.59 | -33.8 ± 15.6 | -35.1 ± 15.8 | 0.08 |
| Aortic max velocity (cm/s) | 105.4 ± 41.9 | 105.3 ± 40.2 | 0.94 | 103.4 ± 33.2 | 103.1 ± 33.1 | 0.83 |
| Aortic min velocity (cm/s) | -77.09 ± 47. | -75.7 ± 45.3 | 0.53 | -64.2 ± 31.6 | -64.8 ± 32.3 | 0.65 |
| Aortic max mean velocity (cm/s) | 40.1 ± 25.1 | 40.67 ± 25.1 | 0.63 | 40.8 ± 21.1 | 41.3 ± 21.3 | 0.57 |
| Aortic max acceleration (cm/s/s) | 0.6 ± 0.2 | 0.6 ± 0.2 | 0.55 | 0.6 ± 0.1 | 0.6 ± 0.2 | 0.18 |
| Aortic min acceleration (cm/s/s) | -0.4 ± 0.1 | -0.4 ± 0.1 | 0.86 | -0.4 ± 0.1 | -0.4 ± 0.1 | 0.23 |

Supplementary Table 9 - Differences in CMR metrics analyzed between hospitalized and propensity-matched controls at baseline and repeat imaging.

| Clinical metric name | Baseline imaging | | | Repeat imaging | | |
| --- | --- | --- | --- | --- | --- | --- |
| Left ventricle structure, function & myocardium measurements | Control | Cases | P-value | Control | Cases | P-value |
| LV end diastolic volume (ml) | 152.1 ±24.6 | 151.2 ±30.7 | 0.88 | 152.3 ±27.4 | 150.7 ±30.6 | 0.82 |
| LV systolic volume (ml) | 89.4 ±15.3 | 91.2±18.8 | 0.66 | 91.7 ±18.6 | 89.2 ±16.1 | 0.54 |
| LV ejection fraction (%) | 59.6 ±4.2 | 59.7 ±6.6 | 0.93 | 61.3 ±5.1 | 59.0 ±6.9 | 0.10 |
| LV mass (g) | 94.4 ± 17.2 | 99.4 ± 22.5 | 0.28 | 94.8 ± 19.5 | 97.4 ± 21.7 | 0.57 |
| LV global longitudinal strain (%) | -17.3 ±2.2 | -17.8 ±2.07 | 0.26 | -17.5 ±2.4 | -16.7 ±2.9 | 0.22 |
| LV global circumferential strain (%) | -18.2 ±2.1 | -17.7±2.8 | 0.34 | -18.3 ±2.4 | -17.1 ±2.8 | 0.06 |
| LV global radial strain (%) | 30.2 ±5.3 | 28.9 ±7.07 | 0.40 | 30.3 ±6.3 | 27.3 ±6.5 | 0.06 |
| Native T1 (ms) | 916.3 ±37.6 | 907.9 ±50.1 | 0.44 | 914.2 ±36.8 | 923.7 ±42.0 | 0.30 |
| RV structure & function |  |  |  |  |  |  |
| RV end diastolic volume (ml) | 165.4 ±33.3 | 160.3 ±35.4 | 0.53 | 165.7 ±33.5 | 160.7 ±35.2 | 0.53 |
| RV systolic volume (ml) | 96.6 ±20.8 | 94.2 ±22.3 | 0.64 | 96.1 ±20.6 | 92.3 ±21.01 | 0.44 |
| RV ejection fraction (%) | 58.4 ±5.3 | 58.8 ±6.6 | 0.77 | 58.1 ±6.1 | 57.6 ±5.4 | 0.69 |
| RV global longitudinal strain (%) | -24.1 ±3.3 | -25.4 ±3.3 | 0.09 | -25.2 ±3.5 | -23.9 ±4.2 | 0.16 |
| RV global circumferential strain (%) | -15.2 ± 2.5 | -14.8 ±3.3 | 0.51 | -15.3 ±2.7 | -14.4 ± 3.1 | 0.16 |
| RV global radial strain (%) | 25.1 ±5.9 | 24.5 ±7.3 | 0.71 | 25.2 ±6.8 | 23.1 ±6.4 | 0.17 |
| Atrial volumes |  |  |  |  |  |  |
| LA maximum volume (ml) | 71.9 ± 23.7 | 75.6 ± 24.1 | 0.50 | 74.6 ± 25.3 | 75.5 ± 20.9 | 0.87 |
| LA emptying fraction (%) | 64.1 ± 10.4 | 66.3 ± 7.2 | 0.31 | 64.6 ± 9.7 | 64.1 ± 9.1 | 0.78 |
| RA maximum volume (ml) | 89.1 ± 25.03 | 83.6 ± 25.2 | 0.35 | 94.5 ± 24.4 | 80.4 ± 24.6 | 0.02 |
| RA emptying fraction (%) | 50.2 ± 8.9 | 53.1 ± 10.7 | 0.21 | 49.4 ± 10.2 | 53.3 ± 11.01 | 0.12 |
| Vascular metrics |  |  |  |  |  |  |
| Ascending aortic strain | 0.0959 ±0.0568 | 0.0812 ±0.0316 | 0.21 | 0.0824 ±0.0356 | 0.0791 ±0.0323 | 0.7 |
| Ascending aorta distensibility (x10^-3^mmHg^-1^) | 0.0018 ±0.0012 | 0.0013 ±0.0006 | 0.04 | 0.0016 ±0.0009 | 0.0013 ±0.0007 | 0.12 |
| Descending aortic strain | 0.1459 ±0.053 | 0.1294 ±0.0393 | 0.14 | 0.1301 ±0.0409 | 0.1242 ±0.0359 | 0.53 |
| Descending aorta distensibility (x10^-3^mmHg^-1^) | 0.0027 ±0.0012 | 0.0021 ±0.0009 | 0.01 | 0.0025 ±0.0011 | 0.002 ±0.0008 | 0.04 |
| Aortic valve metrics |  |  |  |  |  |  |
| Aortic forward flow volume (ml) | 49.4 ± 37.4 | 55.6 ± 33.9 | 0.46 | 54.5 ± 32.4 | 53.6 ± 30.6 | 0.90 |
| Aortic backward volume (ml) | -32.8 ± 37.8 | -28.9 ± 43.3 | 0.68 | -6.1 ± 3.3 | -4.9 ± 2.7 | 0.14 |
| Aortic mean peak gradient (mmHg) | 1.2 ± 0.5 | 1.2 ± 0.5 | 0.86 | 1.1 ± 0.2 | 1.1 ± 0.3 | 0.62 |
| LV – Left ventricle, RV – Right ventricle, LA – Left atrium, RA – Right atrium  Significant p values are highlighted with **bold** text and * next to them  The mean value for each metric and standard deviation are shown. | | | | | | |

Supplementary Table 10 - Differences in the independent ECG leads measured in COVID cases and control cohorts at both baseline and repeat imaging visits

| ECG biomarkers | Baseline imaging | | | Repeat imaging | | |
| --- | --- | --- | --- | --- | --- | --- |
|  | Control | Cases | P-value | Control | Cases | P-value |
| P area I | 159.0 ±103.7 | 165.1 ±98.1 | 0.51 | 158.1 ±89.1 | 162.5 ±88.2 | 0.39 |
| P area II | 263.5 ±149.2 | 272.9 ±151.1 | 0.18 | 266.2 ±141.1 | 268.9 ±142.1 | 0.66 |
| P area V1 | -1.7 ±135.9 | 0.2 ±135.8 | 0.48 | 16.9 ±120.7 | 25.3 ±117.8 | 0.21 |
| P area V2 | 77.2 ±108.7 | 75.5 ±106.6 | 0.67 | 80.5 ±103.4 | 78.9 ±104.0 | 0.96 |
| P area V3 | 166.1 ±85.4 | 168.5 ±83.5 | 0.64 | 175.0 ±82.7 | 175.7 ±80.7 | 0.54 |
| P area V4 | 184.3 ±79.3 | 183.9 ±80.2 | 0.9 | 181.8 ±78.8 | 181.1 ±79.5 | 0.94 |
| P area V5 | 177.1 ±79.04 | 178.1 ±80.1 | 0.70 | 176.9 ±76.1 | 176.8 ±79.2 | 0.86 |
| P area V6 | 166.3 ±76.2 | 168.2 ±77.8 | 0.57 | 167.2 ±73.7 | 167.1 ±78.3 | 0.78 |
| P duration I | 96.3 ±19.6 | 96.3 ±19.3 | 0.68 | 93.2 ± 22.8 | 94.5 ±21.3 | 0.31 |
| P duration II | 98.03 ±18.1 | 97.8 ±18.1 | 0.65 | 94.8 ±20.5 | 95.4 ±20.1 | 0.55 |
| P duration V1 | 64.1 ±31.6 | 65.5 ±31.4 | 0.31 | 64.7 ±30.5 | 63.4 ±30.03 | 0.50 |
| P_duration V2 | 77.2 ±30.8 | 76.05 ±31.6 | 0.40 | 73.2 ±32.7 | 74.6 ±33.5 | 0.22 |
| P duration V3 | 91.2 ±25.0 | 90.9 ±25.1 | 0.52 | 90.9 ±24.0 | 91.1 ±25.1 | 0.64 |
| P duration V4 | 97.1 ±18.5 | 97.0 ±18.1 | 0.64 | 94.9 ±19.7 | 95.5 ±19.0 | 0.70 |
| P duration V5 | 98.17 ± 17.1 | 97.5 ±17.4 | 0.40 | 96.1 ±18.2 | 96.5 ±17.9 | 0.73 |
| P duration V6 | 98.7 ±16.8 | 98.7 ±16.3 | 0.70 | 96.2 ±18.1 | 96.4 ±18.0 | 0.90 |
| P amplitude I | 69.8 ±33.8 | 71.7 ±33.4 | 0.46 | 67.1 ±32.5 | 69.8 ±32.5 | 0.06 |
| P amplitude II | 98.3 ±49.4 | 102.3 ±49.7 | 0.10 | 103.04±46.6 | 103.6 ±48.2 | 0.75 |
| P amplitude V1 | 9.6 ±64.9 | 10.20 ±65.3 | 0.45 | 18.02 ±62.5 | 20.8 ±60.9 | 0.46 |
| P amplitude V2 | 40.5 ±49.8 | 40.9 ±48.6 | 0.86 | 42.8 ±48.1 | 41.6 ±47.7 | 0.78 |
| P amplitude V3 | 72.2 ±27.1 | 72.9 ±26.7 | 0.47 | 73.6 ±25.5 | 74.1 ±26.2 | 0.36 |
| P amplitude V4 | 70.5 ±23.6 | 70.9 ±24.5 | 0.62 | 70.3 ±24.1 | 69.9 ±24.7 | 0.93 |
| P amplitude V5 | 65.2 ±23.4 | 65.9 ±23.8 | 0.43 | 66.3 ±22.3 | 66.3 ±23.8 | 0.80 |
| P amplitude V6 | 60.6 ±22.7 | 60.9 ±24.03 | 0.68 | 62.2 ±22.04 | 62.3 ±24.1 | 0.58 |
| Q area I | 19.9 ±24.4 | 19.2 ±24.5 | 0.39 | 19.7 ±23.9 | 18.2 ±22.6 | 0.24 |
| Q area II | 12.3 ±18.7 | 13.1 ±19.7 | 0.49 | 11.9 ±18.4 | 12.1 ±18.3 | 0.52 |
| Q area V5 | 9.9 ±16.2 | 9.7 ±15.7 | 0.84 | 8.1 ±13.9 | 8.6 ±14.7 | 0.55 |
| Q area V6 | 19.1 ±25.4 | 18.6 ±24.9 | 0.70 | 14.5 ±21.0 | 15.2 ±21.8 | 0.54 |
| Q duration I | 11.5 ±10.4 | 11.02 ±10.4 | 0.21 | 11.2 ±10.1 | 10.7 ±9.7 | 0.25 |
| Q duration II | 7.8 ±9.5 | 7.8 ±9.3 | 0.96 | 7.4 ±9.3 | 7.6 ±9.2 | 0.56 |
| Q duration V5 | 6.7 ±8.7 | 6.6 ±8.6 | 0.83 | 6.3 ±8.6 | 6.2 ±8.3 | 0.86 |
| Q duration V6 | 9.8 ±9.4 | 9.6 ±9.3 | 0.60 | 8.5 ±9.4 | 8.5 ±9.3 | 0.91 |
| Q amplitude I | 38.4 ±44.2 | 36.8 ±42.8 | 0.47 | 37.1 ±41.5 | 34.8 ±39.3 | 0.35 |
| Q amplitude II | 26.4 ±38.3 | 26.6 ±37.3 | 0.65 | 26.08 ±37.2 | 25.7 ±36.2 | 0.83 |
| Q amplitude V5 | 23.1 ±35.6 | 23.6 ±36.4 | 0.94 | 21.3 ±34.7 | 22.07 ±35.5 | 0.71 |
| Q amplitude V6 | 36.9 ±45.1 | 37.7 ±47.2 | 0.95 | 30.4 ±41.8 | 31.5 ±42.5 | 0.60 |
| QRS area I | 721.9 ±492.4 | 757.1 ±495.1 | 0.08 | 759.9 ±446.4 | 792.7 ±437.5 | 0.02 |
| QRS area II | 748.7 ±569.3 | 789.06 ±564.2 | 0.15 | 758.9 ±565.1 | 775.3 ±543.9 | 0.84 |
| QRS area V1 | -846.5 ±611.08 | -895.3 ±611.8 | 0.05 | -848.8 ±609.2 | -876.4 ±626.2 | 0.71 |
| QRS area V2 | -704.3 ±942.2 | -773.1 ±922.5 | 0.11 | -690.0 ±918.08 | -719.1 ±910.1 | 0.89 |
| QRS area V3 | -205.1 ±1108.6 | -224.5 ±1037.6 | 0.57 | 52.08 ±993.9 | 0.7 ±970.7 | 0.13 |
| QRS area V4 | 720.8 ±890.1 | 701.05 ±909.3 | 0.40 | 827.9 ±878.1 | 793.7 ±865.5 | 0.20 |
| QRS area V5 | 1070.8 ±722.6 | 1093.9 ±750.1 | 0.99 | 1052.03 ±756.09 | 1061.7 ±751.4 | 0.79 |
| QRS area V6 | 1078.1 ±578.5 | 1103.7 ±607.5 | 0.63 | 1013.5 ±646.1 | 1038.5 ±666.1 | 0.36 |
| QRS interval I | 38.7 ±5.7 | 39.2 ±5.8 | 0.39 | 38.5.56 | 38.7 ±5.8 | 0.71 |
| QRS interval II | 39.9 ±6.8 | 40.4 ±6.4 | 0.01 | 40.2 ±7.2 | 40.1 ±6.9 | 0.92 |
| QRS interval V1 | 44.3 ±8.5 | 44.6 ±8.3 | 0.30 | 45.1 ±8.7 | 45.3 ±8.8 | 0.67 |
| QRS interval V2 | 46.0 ±10.2 | 47.1 ±9.9 | 0.01 | 47.1 ±10.9 | 47.1 ±10.4 | 0.81 |
| QRS interval V3 | 45.9 ±11.4 | 46.0 ±11.8 | 0.94 | 44.8 ±12.3 | 45.3 ±12.1 | 0.36 |
| QRS interval V4 | 40.7 ±9.4 | 41.04 ±9.8 | 0.76 | 39.9 ±8.8 | 40.1 ±9.2 | 0.97 |
| QRS interval V5 | 38.9 ±6.6 | 39.2 ±6.3 | 0.22 | 38.8 ±6.9 | 38.7 ±6.6 | 0.85 |
| QRS interval V6 | 39.1 ±5.5 | 39.4 ±5.5 | 0.27 | 39.3 ±6.7 | 39.3 ±6.3 | 0.8 |
| R area I | 821.5 ±423.2 | 853.2 ±432.3 | 0.07 | 833.9 ±405.7 | 860.7 ±397.5 | 0.03 |
| R area II | 852.5 ±486.7 | 880.2 ±498.0 | 0.22 | 872.7 ±461.6 | 872.0 ±460.2 | 0.94 |
| R area V1 | 133.4 ±119.9 | 131.1 ±118.9 | 0.70 | 143.3 ±128.7 | 138.7 ±124.1 | 0.61 |
| R area V2 | 377.2 ±327.3 | 353.6 ±304.5 | 0.23 | 355.0±305.0 | 341.5 ±286.6 | 0.46 |
| R area V3 | 719.6 ±520.1 | 725.8 ±514.1 | 0.60 | 817.8 ±532.0 | 796.6 ±521.7 | 0.44 |
| R area V4 | 1203.3 ±609.5 | 1195.0 ±638.2 | 0.56 | 1237.2 ±614.5 | 1225.6 ±635.1 | 0.45 |
| R area V5 | 1343.7 ±587.8 | 1358.6 ±641.6 | 0.83 | 1320.2 ±596.9 | 1327.2 ±620.3 | 0.93 |
| R area V6 | 1218.3 ±515.6 | 1226.6 ±540.2 | 0.78 | 1183.5 ±529.1 | 1200.5 ±541.9 | 0.38 |
| R duration I | 57.4 ±18.8 | 58.1 ±19.3 | 0.65 | 59.8 ±18.5 | 59.3 ±18.5 | 0.62 |
| R duration II | 59.1 ±18.4 | 60.5 ±18.4 | 0.04 | 58.6 ±18.02 | 59.1 ±18.08 | 0.33 |
| R duration V1 | 24.9 ±9.8 | 25.1 ±9.7 | 0.98 | 25.3 ±10.2 | 25.2 ±10.03 | 0.90 |
| R duration V2 | 32.7 ±11.8 | 32.3 ±10.7 | 0.71 | 32.4 ±12.3 | 32.1 ±12.5 | 0.72 |
| R duration V3 | 41.2 ±12.1 | 41.3 ±11.1 | 0.60 | 43.5 ±10.6 | 43.4 ±10.5 | 0.98 |
| R duration V4 | 47.7 ±9.5 | 48.4 ±9.9 | 0.15 | 49.3 ±10.7 | 49.1 ±10.6 | 0.78 |
| R duration V5 | 50.1 ±12.5 | 50.2 ±12.4 | 0.90 | 50.2 ±11.7 | 49.5 ±11.9 | 0.19 |
| R duration V6 | 54.5 ±14.5 | 54.9 ±14.7 | 0.61 | 54.1 ±14.2 | 53.8 ±14.3 | 0.62 |
| R amplitude I | 671.5 ±300.3 | 697.6 ±305.8 | 0.04 | 674.1 ±284.2 | 697.1 ±278.7 | 0.01 |
| R amplitude II | 719.1 ±367.5 | 723.4 ±358.6 | 0.71 | 728.8 ±331.3 | 718.8 ±323.8 | 0.61 |
| R amplitude V1 | 166.4 ±127.6 | 162.3 ±124.7 | 0.52 | 173.7 ±134.3 | 165.9 ±123.1 | 0.48 |
| R amplitude V2 | 407.3 ±304.3 | 386.4 ±286.5 | 0.21 | 390.9 ±296.6 | 377.9 ±285.1 | 0.43 |
| R amplitude V3 | 667.7±425.2 | 666.2 ±416.1 | 0.79 | 739.2 ±429.2 | 729.5 ±433.0 | 0.56 |
| R amplitude V4 | 1084.2 ±511.1 | 1076.3 ±546.9 | 0.48 | 1124.6 ±510.2 | 1108.9 ±542.1 | 0.27 |
| R amplitude V5 | 1223.1 ±470.0 | 1225.1 ±523.0 | 0.73 | 1203.5 ±487.1 | 1205.1 ±510.8 | 0.82 |
| R amplitude V6 | 1069.6 ±394.5 | 1074.5 ±430.1 | 0.97 | 1039.1 ±415.9 | 1052.2 ±428.4 | 0.46 |
| S area I | 38.5 ±65.2 | 42.4 ±67.4 | 0.08 | 26.3 ±47.7 | 32.2 ±51.8 | 0.00 |
| S area II | 67.7 ±103.4 | 65.3 ±103.9 | 0.29 | 75.4 ±114.4 | 67.4 ±104.7 | 0.28 |
| S area V1 | 955.9 ±579.9 | 1006.4 ±585.5 | 0.04 | 946.5 ±578.05 | 967.2 ±614.5 | 0.72 |
| S area V2 | 1104.1 ±732.7 | 1162.7 ±749.4 | 0.10 | 1074.4 ±765.3 | 1078.1 ±793.9 | 0.74 |
| S area V3 | 929.3 ±728.2 | 952.5 ±697.6 | 0.20 | 776.8 ±587.8 | 822.9 ±617.7 | 0.12 |
| S area V4 | 470.1 ±428.7 | 495.3 ±442.7 | 0.24 | 405.5 ±389.7 | 434.6 ±408.3 | 0.12 |
| S area V5 | 243.0 ±261.7 | 257.1 ±275.9 | 0.39 | 248.7 ±271.8 | 267.3 ±287.9 | 0.16 |
| S area V6 | 101.0 ±137.4 | 106.5 ±144.3 | 0.46 | 144.7 ±201.7 | 140.7 ±201.8 | 0.85 |
| S duration I | 15.1 ±19.3 | 16.4 ±19.8 | 0.09 | 13.5 ±18.8 | 14.2 ±18.2 | 0.13 |
| S duration II | 16.7 ±19.4 | 15.9 ±19.5 | 0.25 | 17.4 ±20.1 | 16.2 ±19.1 | 0.21 |
| S duration V1 | 49.8 ±19.4 | 50.9 ±19.1 | 0.19 | 48.4 ±19.6 | 49.4 ±20.03 | 0.14 |
| S duration V2 | 45.5 ±16.6 | 47.2 ±15.7 | 0.03 | 43.6 ±18.5 | 43.9 ±18.7 | 0.73 |
| S duration V3 | 41.1 ±15.1 | 42.1 ±15.4 | 0.18 | 38.3 ±14.8 | 39.1 ±14.7 | 0.33 |
| S duration V4 | 32.7 ±15.4 | 33.4 ±15.6 | 0.24 | 30.9 ±16.6 | 31.6 ±15.8 | 0.23 |
| S duration V5 | 27.4 ±17.5 | 28.1 ±17.6 | 0.24 | 27.4 ±18.2 | 27.9 ±17.3 | 0.40 |
| S duration V6 | 20.3 ±18.9 | 21.1 ±19.7 | 0.44 | 21.8 ±19.7 | 21.9 ±19.8 | 0.99 |
| S amplitude I | 57.4 ±85.7 | 60.8 ±84.5 | 0.13 | 46.5 ±72.7 | 48.5 ±69.0 | 0.12 |
| S amplitude II | 86.4 ±113.9 | 79.7 ±110.8 | 0.15 | 97.4 ±128.7 | 85.8 ±115.5 | 0.13 |
| S amplitude V1 | 705.7 ±380.4 | 732.9 ±372.1 | 0.05 | 686.2 ±373.5 | 690.5 ±385.8 | 0.84 |
| S amplitude V2 | 849.1 ±464.5 | 879.2 ±460.6 | 0.14 | 827.9 ±511.6 | 821.4 ±519.7 | 0.52 |
| S amplitude V3 | 772.7 ±474.4 | 776.8 ±448.9 | 0.47 | 698.5 ±418.9 | 711.8 ±421.4 | 0.43 |
| S amplitude V4 | 468.6 ±347.8 | 472.5 ±331.1 | 0.43 | 409.7 ±321.6 | 429.1 ±323.7 | 0.14 |
| S amplitude V5 | 251.5 ±224.7 | 256.9 ±228.1 | 0.64 | 252.2 ±225.9 | 270.9 ±237.8 | 0.10 |
| S amplitude V6 | 111.6 ±131.4 | 116.1 ±134.6 | 0.50 | 153.4 ±190.1 | 151.4 ±190.7 | 0.88 |
| T area I | 1112.5 ±705.0 | 1172.2 ±675.3 | 0.17 | 1153.9 ±598.5 | 1167.9 ±620.9 | 0.63 |
| T area II | 1403.1 ±791.5 | 1364.2 ±750.4 | 0.28 | 1458.6 ±722.5 | 1414.4 ±734.2 | 0.20 |
| T area V1 | 54.04 ±989.6 | 22.0 ±995.2 | 0.47 | 234.07 ±1005.3 | 205.1 ±966.7 | 0.76 |
| T area V2 | 1759.9 ±1445.6 | 1698.7 ±1513.9 | 0.15 | 1865.8 ±1563.5 | 1727.2 ±1549.8 | 0.03 |
| T area V3 | 2379.2 ±1456.3 | 2242.3 ±1429.6 | 0.03 | 2503.7 ±1418.7 | 2374.5 ±1462.5 | 0.01 |
| T area V4 | 2272.5 ±1268.6 | 2136.8 ±1241.2 | 0.02 | 2294.9 ±1319.3 | 2163.9 ±1258.6 | 0.02 |
| T area V5 | 1895.9 ±1071.1 | 1820.08 ±1040.3 | 0.15 | 2004.1 ±1063.5 | 1898.2 ±1077.8 | 0.02 |
| T area V6 | 1489.0 ±848.1 | 1446.4 ±806.8 | 0.40 | 1664.1 ±882.0 | 1598.7 ±893.4 | 0.10 |
| T duration I | 200.7 ±28.0 | 201.8 ±27.7 | 0.43 | 205.1 ±25.8 | 207.5 ±25.5 | 0.05 |
| T duration II | 201.7 ±26.4 | 201.9 ±28.1 | 0.80 | 206.5 ±25.3 | 207.4 ±26.0 | 0.46 |
| T duration V1 | 162.0 ±61.1 | 166.9 ±58.8 | 0.13 | 170.09 ±58.2 | 168.9 ±59.2 | 0.72 |
| T duration V2 | 189.5 ±37.7 | 189.1 ±39.09 | 0.81 | 197.7 ±34.8 | 198.9 ±35.3 | 0.48 |
| T duration V3 | 202.05 ±26.2 | 201.01 ±29.6 | 0.92 | 207.7 ±23.7 | 208.4 ±25.7 | 0.37 |
| T duration V4 | 202.9 ±24.6 | 202.9 ±26.1 | 0.80 | 207.5 ±23.3 | 208.6 ±25.3 | 0.26 |
| T duration V5 | 201.3 ±25.5 | 203.08 ±26.4 | 0.13 | 207.4 ±24.05 | 208.1 ±24.4 | 0.59 |
| T duration V6 | 199.1 ±27.51 | 200.5 ±29.4 | 0.14 | 206.4 ±24.5 | 207.04 ±26.2 | 0.34 |
| T end I | 26.8 ±32.9 | 28.03 ±30.7 | 0.68 | 26.7 ±29.0 | 26.1 ±29.8 | 0.60 |
| T end II | 38.1 ±38.1 | 39.01 ±39.6 | 0.53 | 36.3 ±35.7 | 37.01 ±36.9 | 0.48 |
| T end V1 | -11.07 ±35.6 | -12.5 ±36.6 | 0.54 | -3.7 ±34.2 | -3.6 ±31.8 | 0.67 |
| T end V2 | 18.1 ±41.7 | 15.8 ±40.2 | 0.38 | 24.9 ±38.9 | 22.4 ±36.7 | 0.18 |
| T end V3 | 41.8 ±39.8 | 39.8 ±36.4 | 0.25 | 50.5 ±37.3 | 47.1 ±35.9 | 0.05 |
| T end V4 | 43.2 ±37.8 | 41.9 ±36.8 | 0.27 | 47.7 ±36.8 | 45.9 ±33.0 | 0.30 |
| T end V5 | 35.5 ±34.0 | 35.2 ±32.4 | 0.58 | 42.3 ±31.4 | 39.9 ±30.8 | 0.14 |
| T end V6 | 27.2 ±31.6 | 27.7 ±31.8 | 0.62 | 34.09 ±31.1 | 33.0 ±28.6 | 0.80 |
| T amplitude I | 202.1 ±123.4 | 211.1 ±118.2 | 0.19 | 208.1 ±103.8 | 211.4 ±105.1 | 0.61 |
| T amplitude II | 247.8 ±133.6 | 240.7 ±130.4 | 0.32 | 257.9 ±123.8 | 247.4 ±126.3 | 0.07 |
| T amplitude V1 | 5.6 ±180.1 | 2.6 ±181.4 | 0.67 | 35.7 ±179.3 | 32.5 ±175.1 | 0.76 |
| T amplitude V2 | 300.6 ±245.3 | 289.08 ±256.1 | 0.14 | 309.6 ±265.5 | 287.1 ±262.4 | 0.03 |
| T amplitude V3 | 397.2 ±249.5 | 374.4 ±242.3 | 0.05 | 407.9 ±237.9 | 388.4 ±245.1 | 0.02 |
| T amplitude V4 | 391.5 ±221.0 | 368.6 ±211.5 | 0.03 | 388.2 ±221.7 | 364.7 ±214.3 | 0.01 |
| T amplitude V5 | 338.2 ±186.1 | 329.03 ±185.6 | 0.20 | 350.8 ±183.4 | 329.8 ±185.3 | 0.01 |
| T amplitude V6 | 273.2 ±148.7 | 268.1 ±146.8 | 0.46 | 296.5 ±154.4 | 285.17 ±161.5 | 0.06 |

Supplementary Table 11 - Logistic regression results for CMR metrics

| Clinical metric name | Beta | P-value |
| --- | --- | --- |
| LV structure, function & myocardium measurements |  |  |
| LV end diastolic volume (EDV) (ml) | 0.0007 | 0.64 |
| LV systolic volume (SV) (ml) | -0.0011 | 0.68 |
| LV ejection fraction (EF) (%) | -0.0125 | 0.05 |
| LV mass (g) | 0.0050 | 0.12 |
| LV global longitudinal strain (GLS) (%) | 0.0407 | 0.03* |
| LV global circumferential strain (GCS) (%) | 0.0543 | 0.003* |
| LV global radial strain (GRS) (%) | -0.0249 | 0.002* |
| Native T1 (ms) | -0.0002 | 0.60 |
| RV structure & function |  |  |
| RV end diastolic volume (EDV) ml | 0.0008 | 0.61 |
| RV systolic volume (SV) ml | -0.0001 | 0.97 |
| RV ejection fraction (EF) % | -0.0079 | 0.22 |
| RV global longitudinal strain (GLS) % | 0.0031 | 0.79 |
| RV global circumferential strain (GCS) % | 0.0154 | 0.30 |
| RV global radial strain (GRS) % | -0.0064 | 0.35 |
| Atrial volumes |  |  |
| LA maximum volume (ml) | -0.0021 | 0.25 |
| LA emptying fraction (%) | 0.0009 | 0.83 |
| RA maximum volume (ml) | -0.0016 | 0.36 |
| RA emptying fraction (%) | 0.0030 | 0.52 |
| Vascular metrics |  |  |
| Ascending aortic strain | -1.1325 | 0.24 |
| Ascending aorta distensibility | -29.1518 | 0.52 |
| Descending aortic strain | -1.2407 | 0.20 |
| Descending aorta distensibility | -44.8797 | 0.29 |
| Aortic valve volumes |  |  |
| Aortic forward flow volume | -0.0001 | 0.97 |
| Aortic backward volume | 0.0011 | 0.39 |
| Aortic mean peak gradient | -0.1017 | 0.34 |
| The logistic regression models are adjusted for age, sex, ethnicity, deprivation, BMI, smoking, diabetes, hypertension, hypercholesterolemia and prevalent myocardial infarction. Beta co-efficient and p values for logistic regression analysis are detailed here and demonstrated Figure 2  LV – Left ventricle, RV – Right ventricle, LA – Left atrium, RA – Right atrium  Significant p values are highlighted with **bold** text and * next to them | | |

Supplementary Table 12 - Association of CMR metrics at baseline and incident COVID-19.

| Clinical metric name |  |  | | |
| --- | --- | --- | --- | --- |
| LV structure & function measurements | OR | CI[0.025] | Cl[0.975] | P-value |
| LV end systolic volume (ml) | 1 | 1 | 1.01 | 0.21 |
| LV cardiac output (L/min) | 0.97 | 0.89 | 1.05 | 0.45 |
| LV mass in systole (g) | 1 | 1 | 1.01 | 0.15 |
| Diastolic total peak wall thickness (mm) | 1.05 | 0.98 | 1.14 | 0.18 |
| Total peak wall thickness (mm) | 1.05 | 0.98 | 1.14 | 0.18 |
| LV MAPSE lateral (mm) | 0.98 | 0.95 | 1 | 0.09 |
| LV MAPSE septal (mm) | 0.98 | 0.95 | 1.01 | 0.11 |
| LV TAPSE (mm) | 1 | 0.98 | 1.01 | 0.61 |
| 2ch- LV long axis strain (%) | 1.02 | 1 | 1.05 | 0.11 |
| 2ch – LV long axis difference (mm) | 1.01 | 0.99 | 1.04 | 0.28 |
| 2ch – LVAV junction strain (%) | 1.02 | 1 | 1.05 | 0.11 |
| 2ch – LVAV junction difference (mm) | 1.02 | 0.99 | 1.04 | 0.2 |
| 2ch LA long axis strain (%) | 1 | 1 | 1.01 | 0.47 |
| 2ch LA long axis differenece (cm) | 1.01 | 0.99 | 1.02 | 0.5 |
| 2ch LAAV junction strain (%) | 1 | 1 | 1.01 | 0.7 |
| 2ch LAAV junction difference (mm) | 1 | 0.99 | 1.02 | 0.62 |
| 4ch LV long axis strain (%) | 1.02 | 0.99 | 1.04 | 0.14 |
| 4ch LV long axis difference (mm) | 1.02 | 0.99 | 1.04 | 0.18 |
| 4ch LVAV junction strain (%) | 1.02 | 1 | 1.05 | 0.1 |
| 4ch LVAV junction difference (mm) | 1.02 | 0.99 | 1.04 | 0.15 |
| 4ch LA long axis strain (%) | 1 | 0.99 | 1.01 | 0.82 |
| 4ch LA long axis difference (mm) | 1 | 0.98 | 1.02 | 0.87 |
| 4ch LAAV junction strain (%) | 1 | 0.99 | 1.01 | 0.87 |
| 4ch LAAV junction difference (mm) | 1 | 0.98 | 1.02 | 0.99 |
| 4ch RA long axis Strain (%) | 1 | 0.99 | 1.01 | 0.5 |
| 4ch RA long axis difference (mm) | 1 | 0.98 | 1.03 | 0.9 |
| 4ch RAAV junction strain (%) | 1 | 0.99 | 1.01 | 0.83 |
| 4ch RAAV junction difference (mm) | 1 | 0.98 | 1.03 | 0.72 |
| 2ch-4ch average long axis strain (%) | 1.02 | 0.99 | 1.05 | 0.24 |
| 2ch-4ch average long axis difference (mm) | 1.02 | 0.99 | 1.04 | 0.28 |
| 2ch-4ch average LVAV junction strain (%) | 1.02 | 0.99 | 1.05 | 0.16 |
| 2ch-4ch average LVAV junction difference (mm) | 1.02 | 0.99 | 1.05 | 0.2 |
| 2ch-4ch average LAX strain (%) | 1 | 0.99 | 1.01 | 0.81 |
| 2ch-4ch average LA LAX difference (mm) | 1.02 | 0.99 | 1.04 | 0.2 |
| 2ch-4ch average LAAV junction strain (%) | 1 | 0.99 | 1.01 | 0.78 |
| RV structure & function |  |  |  |  |
| RV end systolic volume | 1 | 1 | 1.01 | 0.34 |
| RV cardiac output (L/min) | 1.02 | 0.95 | 1.11 | 0.54 |
| RV mass in diastole (g) | 1 | 0.98 | 1.01 | 0.92 |
| RV mass in systole (g) | 1.01 | 0.99 | 1.04 | 0.23 |
| Vascular metrics |  |  |  |  |
| Mean systolic ascending aortic area (mm^2^) | 0.99 | 0.99 | 1 | 0.19 |
| Mean diastolic ascending aortic area (mm^2^) | 0.99 | 0.99 | 1 | 0.35 |
| Mean systolic descending aortic area (mm^2^) | 1 | 0.99 | 1 | 0.31 |
| Mean diastolic descending area (mm^2^) | 1 | 0.99 | 1 | 0.19 |
| Aortic valve volumes |  |  |  |  |
| LV peak ejection rate (ml/s) | 1 | 1 | 1 | 0.48 |
| LV peak filling rate (ml/s) | 1 | 1 | 1 | 0.66 |
| RV peak ejection rate (ml/s) | 1 | 1 | 1 | 0.16 |
| RV peak filling rate (ml/s) | 1 | 1 | 1 | 0.69 |
| Aortic total volume (ml) | 1 | 1 | 1 | 0.67 |
| Aortic regurgitant fraction (%) | 1.02 | 0.99 | 1.04 | 0.17 |
| Aortic net positive volume (ml) | 1 | 1 | 1 | 0.79 |
| Aortic net negative volume (ml) | 1 | 1 | 1 | 0.39 |
| Aortic max pressure gradient (mmHg) | 0.96 | 0.93 | 1 | 0.07 |
| Aortic max flow (ml/s) | 1 | 1 | 1 | 0.77 |
| Aortic min flow (ml/s) | 1 | 1 | 1 | 0.39 |
| Aortic max velocity (cm/s) | 1 | 1 | 1 | 0.62 |
| Aortic min velocity (cm/s) | 1 | 1 | 1 | 0.15 |
| Aortic max mean velocity (cm/s) | 1 | 1 | 1 | 0.58 |
| Aortic max acceleration (cm/s/s) | 1.1 | 0.74 | 1.63 | 0.65 |
| Aortic min acceleration (cm/s/s) | 1.24 | 0.7 | 2.21 | 0.46 |
| Aortic mean peak gradient (mmHg-1) | 0.90 | 0.73 | 1.1 | 0.34 |
| Aortic backward volume (ml) | 1.0 | 0.99 | 1.0 | 0.39 |
| Aortic forward flow volume (ml) | 0.99 | 1.0 | 1.0 | 0.97 |
| Descending aorta distensibility (x10-3mmHg-1) | 3.23E-20 | 1.85E-56 | 5.64E+16 | 0.29 |
| Descending aortic strain (mm) | 0.2 | 0.04 | 1.9 | 0.19 |
| Ascending aorta distensibility (x10-3mmHg-1) | 2.19E-13 | 5.01E-52 | 9.54E+25 | 0.52 |
| Ascending aortic strain (mm) | 0.3 | 0.049 | 2.094 | 0.23 |
| Results for CMR metrics assessed using logistic regression analysis for association with incident COVID-19. Beta co-efficient values, odds ratio [95% Confidence interval] and associated p values are shown. | | | | |

Supplementary Table 13 – Association between lead-specific ECG biomarkers and incident COVID-19

| ECG biomarker | Odds Ratio | 95% confidence interval | P-value |
| --- | --- | --- | --- |
| P area I | 1.00 | 1.00 -1.00 | 0.45 |
| P area II | 1.00 | 1.00 -1.00 | 0.35 |
| P area V1 | 1.00 | 1.00 -1.00 | 0.80 |
| P area V2 | 1.00 | 1.00 -1.00 | 0.63 |
| P area V3 | 1.00 | 1.00 -1.00 | 0.43 |
| P area V4 | 1.00 | 1.00 -1.00 | 0.95 |
| P area V5 | 1.00 | 1.00 -1.00 | 0.81 |
| P area V6 | 1.00 | 1.00 -1.00 | 0.64 |
| P duration I | 1.00 | 1.00 -1.00 | 0.84 |
| P duration II | 1.00 | 1.00 -1.00 | 0.58 |
| P duration V1 | 1.00 | 1.00 -1.00 | 0.10 |
| P duration V2 | 1.00 | 1.00 -1.00 | 0.40 |
| P duration V3 | 1.00 | 1.00 -1.00 | 0.62 |
| P duration V4 | 1.00 | 1.00 -1.00 | 0.72 |
| P duration V5 | 1.00 | 1.00 -1.00 | 0.37 |
| P duration V6 | 1.00 | 1.00 -1.00 | 0.71 |
| P amplitude I | 1.00 | 1.00 -1.00 | 0.42 |
| P amplitude II | 1.00 | 1.00 -1.00 | 0.23 |
| P amplitude V1 | 1.00 | 1.00 -1.00 | 0.68 |
| P amplitude V2 | 1.00 | 1.00 -1.00 | 0.85 |
| P amplitude V3 | 1.00 | 1.00 -1.00 | 0.43 |
| P amplitude V4 | 1.00 | 1.00 -1.00 | 0.70 |
| P amplitude V5 | 1.00 | 1.00 -1.00 | 0.53 |
| P amplitude V6 | 1.00 | 1.00 -1.00 | 0.87 |
| Q area I | 1.00 | 1.00 -1.00 | 0.32 |
| Q area II | 1.00 | 1.00 -1.00 | 0.62 |
| Q area V5 | 1.00 | 1.00 -1.00 | 0.68 |
| Q area V6 | 1.00 | 1.00 -1.00 | 0.68 |
| Q duration I | 1.00 | 1.00 -1.00 | 0.24 |
| Q duration II | 1.00 | 1.00 -1.00 | 0.63 |
| Q duration V5 | 1.00 | 1.00 -1.00 | 0.62 |
| Q duration V6 | 1.00 | 1.00 -1.00 | 0.68 |
| Q amplitude I | 1.00 | 1.00 -1.00 | 0.22 |
| Q amplitude II | 1.00 | 1.00 -1.00 | 0.81 |
| Q amplitude V5 | 1.00 | 1.00 -1.00 | 0.93 |
| Q amplitude V6 | 1.00 | 1.00 -1.00 | 0.76 |
| QRS area I | 1.00 | 1.00 -1.00 | 0.59 |
| QRS area II | 1.00 | 1.00 -1.00 | 0.24 |
| QRS area V1 | 1.00 | 1.00 -1.00 | 0.18 |
| QRS area V2 | 1.00 | 1.00 -1.00 | 0.16 |
| QRS area V3 | 1.00 | 1.00 -1.00 | 0.64 |
| QRS area V4 | 1.00 | 1.00 -1.00 | 0.68 |
| QRS area V5 | 1.00 | 1.00 -1.00 | 0.45 |
| QRS area V6 | 1.00 | 1.00 -1.00 | 0.35 |
| QRS interval I | 1.00 | 1.00 -1.00 | 0.45 |
| QRS interval II | 1.00 | 1.00 -1.00 | 0.24 |
| QRS interval V1 | 1.00 | 1.00 -1.00 | 0.81 |
| QRS interval V2 | 1.00 | 1.00 -1.00 | 0.02 |
| QRS interval V3 | 1.00 | 1.00 -1.00 | 0.81 |
| QRS interval V4 | 1.00 | 1.00 -1.00 | 0.96 |
| QRS interval V5 | 1.00 | 1.00 -1.00 | 0.86 |
| QRS interval V6 | 1.00 | 1.00 -1.00 | 0.71 |
| R area I | 1.00 | 1.00 -1.00 | 0.58 |
| R area II | 1.00 | 1.00 -1.00 | 0.41 |
| R area V1 | 1.00 | 1.00 -1.00 | 0.48 |
| R area V2 | 1.00 | 1.00 -1.00 | 0.05 |
| R area V3 | 1.00 | 1.00 -1.00 | 0.90 |
| R area V4 | 1.00 | 1.00 -1.00 | 0.81 |
| R area V5 | 1.00 | 1.00 -1.00 | 0.51 |
| R area V6 | 1.00 | 1.00 -1.00 | 0.74 |
| R duration I | 1.00 | 1.00 -1.00 | 0.90 |
| R duration II | 1.00 | 1.00 -1.00 | 0.18 |
| R duration V1 | 1.00 | 1.00 -1.00 | 0.52 |
| R duration V2 | 1.00 | 1.00 -1.00 | 0.33 |
| R duration V3 | 1.00 | 1.00 -1.00 | 0.83 |
| R duration V4 | 1.00 | 1.00 -1.00 | 0.39 |
| R duration V5 | 1.00 | 1.00 -1.00 | 0.93 |
| R duration V6 | 1.00 | 1.00 -1.00 | 0.96 |
| R amplitude I | 1.00 | 1.00 -1.00 | 0.45 |
| R amplitude II | 1.00 | 1.00 -1.00 | 0.96 |
| R amplitude V1 | 1.00 | 1.00 -1.00 | 0.27 |
| R amplitude V2 | 1.00 | 1.00 -1.00 | 0.04 |
| R amplitude V3 | 1.00 | 1.00 -1.00 | 0.93 |
| R amplitude V4 | 1.00 | 1.00 -1.00 | 0.90 |
| R amplitude V5 | 1.00 | 1.00 -1.00 | 0.68 |
| R amplitude V6 | 1.00 | 1.00 -1.00 | 0.68 |
| S area I | 1.00 | 1.00 -1.00 | 0.25 |
| S area II | 1.00 | 1.00 -1.00 | 0.57 |
| S area V1 | 1.00 | 1.00 -1.00 | 0.16 |
| S area V2 | 1.00 | 1.00 -1.00 | 0.15 |
| S area V3 | 1.00 | 1.00 -1.00 | 0.42 |
| S area V4 | 1.00 | 1.00 -1.00 | 0.26 |
| S area V5 | 1.00 | 1.00 -1.00 | 0.28 |
| S area V6 | 1.00 | 1.00 -1.00 | 0.46 |
| S duration I | 1.00 | 1.00 -1.00 | 0.06 |
| S duration II | 1.00 | 1.00 -1.00 | 0.43 |
| S duration V1 | 1.00 | 1.00 -1.00 | 0.26 |
| S duration V2 | 1.00 | 1.00 -1.00 | 0.06 |
| S duration V3 | 1.00 | 1.00 -1.00 | 0.13 |
| S duration V4 | 1.00 | 1.00 -1.00 | 0.35 |
| S duration V5 | 1.00 | 1.00 -1.00 | 0.35 |
| S duration V6 | 1.00 | 1.00 -1.00 | 0.26 |
| S amplitude I | 1.00 | 1.00 -1.00 | 0.28 |
| S amplitude II | 1.00 | 1.00 -1.00 | 0.28 |
| S amplitude V1 | 1.00 | 1.00 -1.00 | 0.29 |
| S amplitude V2 | 1.00 | 1.00 -1.00 | 0.29 |
| S amplitude V3 | 1.00 | 1.00 -1.00 | 0.73 |
| S amplitude V4 | 1.00 | 1.00 -1.00 | 0.74 |
| S amplitude V5 | 1.00 | 1.00 -1.00 | 0.53 |
| S amplitude V6 | 1.00 | 1.00 -1.00 | 0.40 |
| T area I | 1.00 | 1.00 -1.00 | 0.19 |
| T area II | 1.00 | 1.00 -1.00 | 0.17 |
| T area V1 | 1.00 | 1.00 -1.00 | 0.37 |
| T area V2 | 1.00 | 1.00 -1.00 | 0.32 |
| T area V3 | 1.00 | 1.00 -1.00 | 0.11 |
| T area V4 | 1.00 | 1.00 -1.00 | 0.04 |
| T area V5 | 1.00 | 1.00 -1.00 | 0.28 |
| T area V6 | 1.00 | 1.00 -1.00 | 0.43 |
| T duration I | 1.00 | 1.00 -1.00 | 0.88 |
| T duration II | 1.00 | 1.00 -1.00 | 0.62 |
| T duration V1 | 1.00 | 1.00 -1.00 | 0.17 |
| T duration V2 | 1.00 | 1.00 -1.00 | 0.22 |
| T duration V3 | 1.00 | 1.00 -1.00 | 0.31 |
| T duration V4 | 1.00 | 1.00 -1.00 | 0.63 |
| T duration V5 | 1.00 | 1.00 -1.00 | 0.52 |
| T duration V6 | 1.00 | 1.00 -1.00 | 0.67 |
| T end I | 1.00 | 1.00 -1.00 | 0.58 |
| T end II | 1.00 | 1.00 -1.00 | 0.69 |
| T end V1 | 1.00 | 1.00 -1.00 | 0.43 |
| T end V2 | 1.00 | 1.00 -1.00 | 0.26 |
| T end V3 | 1.00 | 1.00 -1.00 | 0.69 |
| T end V4 | 1.00 | 1.00 -1.00 | 0.87 |
| T end V5 | 1.00 | 1.00 -1.00 | 0.70 |
| T end V6 | 1.00 | 1.00 -1.00 | 0.66 |
| T amplitude I | 1.00 | 1.00 -1.00 | 0.23 |
| T amplitude II | 1.00 | 1.00 -1.00 | 0.20 |
| T amplitude V1 | 1.00 | 1.00 -1.00 | 0.56 |
| T amplitude V2 | 1.00 | 1.00 -1.00 | 0.30 |
| T amplitude V3 | 1.00 | 1.00 -1.00 | 0.11 |
| T amplitude V4 | 1.00 | 1.00 -1.00 | 0.04 |
| T amplitude V5 | 1.00 | 1.00 -1.00 | 0.55 |
| T amplitude V6 | 1.00 | 1.00 -1.00 | 0.68 |
| Results for lead specific ECG biomarkers using logistic regression analysis for association with incident COVID-19. Odds ratio, 95% Confidence interval and associated P-values are shown. | | | |

Supplementary Table 14 – Interval change in CMR metrics from baseline to repeat imaging (remaining results)

| Clinical metric name |  | | |
| --- | --- | --- | --- |
| LV structure & function measurements | Control | Cases | P-value |
| LV end systolic volume (ml) | 6.09e-15±9.3 | 2.23e-15±9.1 | 1 |
| LV cardiac output (L/min) | -8.45e-16±0.8 | -1.69e-16±0.8 | 0.95 |
| LV mass in systole (g) | 1.17e-14±9.7 | -8.94e-15±8.9 | 0.91 |
| Diastolic total peak wall thickness (mm) | -1.58e-15±0.7 | -1.07e-15±0.8 | 0.89 |
| Total peak wall thickness (mm) | -1.58e-15±0.7 | -1.07e-15±0.8 | 0.89 |
| LV MAPSE lateral (mm) | -1.81e-15±2.6 | -5.21e-16±2.7 | 0.92 |
| LV MAPSE septal (mm) | 1.28e-15±2.7 | 1.85e-16±2.7 | 0.94 |
| LV TAPSE (mm) | -7.06e-15±3.9 | -1.34e-15±4.1 | 0.67 |
| 2ch- LV long axis strain (%) | -8.92e-16±2.6 | -7.8e-16±2.8 | 0.95 |
| 2ch – LV long axis difference (mm) | -2.44e-15±2.6 | -9.07e-16±2.8 | 0.96 |
| 2ch – LVAV junction strain (%) | 3.69e-16±2.4 | -1.88e-16±2.6 | 0.92 |
| 2ch – LVAV junction difference (mm) | 1.25e-15±2.5 | 1.84e-15±2.7 | 0.95 |
| 2ch LA long axis strain (%) | -4.78e-16±23.4 | 7.81e-16±22.5 | 0.53 |
| 2ch LA long axis differenece (cm) | -8.94e-16±4.6 | 1.63e-15±4.3 | 0.73 |
| 2ch LAAV junction strain (%) | 8.2e-15±17.4 | -8.1e-15±16.4 | 0.32 |
| 2ch LAAV junction difference (mm) | -4.27e-16±4.3 | -1.76e-15±4.05 | 0.67 |
| 4ch LV long axis strain (%) | 2.06e-15±3.1 | -4.67e-15±3.2 | 0.74 |
| 4ch LV long axis difference (mm) | 9.31e-16±2.8 | 6.85e-16±3.1 | 0.63 |
| 4ch LVAV junction strain (%) | -2e-15±2.9 | -6.38e-16±3.07 | 0.74 |
| 4ch LVAV junction difference (mm) | -1.51e-15±2.7 | -3.98e-16±2.9 | 0.65 |
| 4ch LA long axis strain (%) | 3.2e-15±9.2 | 1.75e-15±9.3 | 0.80 |
| 4ch LA long axis difference (mm) | 1.19e-15±3.4 | -3.96e-17±3.7 | 0.84 |
| 4ch LAAV junction strain (%) | -1.37e-15±8.5 | 4.79e-16±8.4 | 0.67 |
| 4ch LAAV junction difference (mm) | -2.32e-16±3.4 | -2.09e-15±3.6 | 0.94 |
| 4ch RA long axis strain (%) | -2.12e-15±9.7 | -4.74e-15±9.9 | 0.94 |
| 4ch RA long axis difference (mm) | 1.72e-15±3.2 | -5.61e-16±3.3 | 0.89 |
| 4ch RAAV junction strain (%) | 4.22e-15±8.5 | -7.16e-16±8.4 | 0.72 |
| 4ch RAAV junction difference (mm) | -1.2e-15±3.05 | -1.3e-15±3.13 | 0.94 |
| 2ch-4ch average long axis strain (%) | -2.44e-16±2.3 | 1.39e-15±2.5 | 0.68 |
| 2ch-4ch average long axis difference (mm) | 1.97e-15±2.2 | -1.4e-15±2.4 | 0.57 |
| 2ch-4ch average LVAV junction strain (%) | 1.05e-15±2.1 | -1.4e-15±2.4 | 0.68 |
| 2ch-4ch average LVAV junction difference (mm) | 9.44e-16±2.1 | 2.43e-15±2.4 | 0.47 |
| 2ch-4ch average LAX strain (%) | 3.01e-15±14.4 | -3.39e-15±13.9 | 0.92 |
| 2ch-4ch average LA LAX difference (mm) | -1.35e-16±3.1 | 1.15e-15±3.1 | 0.80 |
| 2ch-4ch average LAAV junction Strain (%) | 6.09e-15±9.3 | 2.23e-15±9.1 | 1 |
| RV structure & function |  |  |  |
| RV end systolic volume | 2.11e-15±8.1 | 1.35e-15±8.5 | 0.77 |
| RV cardiac output (L/min) | -2.57e-16±0.9 | 2.08e-16±0.8 | 0.66 |
| RV mass in diastole (g) | 5.95e-16±4.0 | -8.68e-15±3.9 | 0.78 |
| RV mass in systole (g) | 3.7e-15±2.5 | -1.66e-15±2.7 | 0.61 |
| Vascular metrics |  |  |  |
| Mean systolic ascending aortic area (mm^2^) | -1.0e-13 ±47.2 | -9.35e-15 ±46.5 | 0.98 |
| Mean diastolic ascending aortic area (mm^2^) | -8.14e-14 ±51.01 | -4.38e-14 ±48.2 | 0.93 |
| Mean systolic descending aortic area (mm^2^) | -5.42e-14 ±28.8 | 1.26e-13 ±31.3 | 0.84 |
| Mean diastolic descending area (mm^2^) | 8.68e-15 ±29.5 | -2.60e-14 ±30.6 | 0.84 |
| Aortic valve volumes |  |  |  |
| LV peak ejection rate (ml/s) | -1.37e-13±124.01 | 3.78e-14±129.4 | 0.95 |
| LV peak filling rate (ml/s) | -5.17e-14±128.5 | 4.92e-14±129.6 | 0.96 |
| RV peak ejection rate (ml/s) | -6.55e-14±137.6 | 3.97e-14±147.4 | 0.84 |
| RV peak filling rate (ml/s) | 3.01e-14±152.3 | -1.42e-14±162.9 | 0.74 |
| Aortic total volume (ml) | -1.92e-15±14.8 | 7.02e-15±15.6 | 0.52 |
| Aortic regurgitant fraction (%) | -1.96e-16±3.9 | 6.54e-16±4.07 | 0.92 |
| Aortic net positive volume (ml) | 1.47e-14±33.9 | -3.65e-16±33.03 | 0.59 |
| Aortic net negative volume (ml) | -8.46e-16±14.1 | 2.63e-15±14.9 | 0.42 |
| Aortic max pressure gradient (mmHg) | -6.72e-16±1.9 | 7.85e-16±1.8 | 0.88 |
| Aortic max flow (ml/s) | 4.32e-15±138.03 | 1.96e-14±136.06 | 0.54 |
| Aortic min flow (ml/s) | -5.97e-16±15.2 | -2.22e-15±15.6 | 0.96 |
| Aortic max velocity (cm/s) | -1.32e-15±31.6 | -8.58e-15±31.4 | 0.65 |
| Aortic min velocity (cm/s) | 4.2e-15±30.5 | -1.06e-14±31.7 | 0.51 |
| Aortic max mean velocity (cm/s) | -3.44e-15±19.7 | -3.43e-15±19.5 | 0.75 |
| Aortic max acceleration (cm/s/s) | 1.16e-16±0.1 | 2.87e-17±0.1 | 1 |
| Aortic min acceleration (cm/s/s) | 2.75e-17±0.1 | 1.83e-17±0.1 | 0.92 |
| Remaining results for residuals acting as standardized change scores, which were calculated by regressing the CMR values from the repeat imaging visit on those from baseline visit. Main results shown in Table 4 in manuscript. | | | |

Supplementary Table 15 – Interval change in ECG biomarkers from baseline to repeat

| ECG biomarker | Control | Cases | P-value |
| --- | --- | --- | --- |
| Atrialrate | 2.96E-15 ±6.6 | 7.40E-16±7.3 | 6.4E-01 |
| P area I | 2.02E-16 ±80.2 | -5.48E-15±80.2 | 7.7E-01 |
| P area II | 2.61E-14 ±120.5 | 3.85E-15 ±123.5 | 6.2E-01 |
| P area V1 | -5.05E-16 ±115.5 | -5.69E-16 ±106.9 | 3.5E-01 |
| P area V2 | -2.51E-16 ±99.4 | 6.02E-15 ±95.6 | 5.8E-01 |
| P area V3 | -8.44E-15 ±74.9 | -3.99E-16 ±74.0 | 6.4E-01 |
| P area V4 | -1.80E-14 ±66.4 | 2.32E-1 ±68.6 | 7.8E-01 |
| P area V5 | -1.49E-14 ±65.9 | -1.35E-14 ±69.3 | 6.4E-01 |
| P area V6 | -1.85E-14 ±64.0 | 6.82E-15 ±68.3 | 6.8E-01 |
| P duration I | -6.22E-16 ±21.1 | 1.03E-14 ±20.5 | 7.7E-01 |
| P duration II | 4.00E-15 ±19.1 | -3.94E-15 ±19.3 | 9.4E-01 |
| P duration V1 | -5.44E-15 ±30.3 | -5.34E-17 ±29.5 | 7.9E-01 |
| P duration V2 | -6.20E-15 ±32.1 | -1.18E-14 ±33.2 | 8.0E-01 |
| P duration V3 | 8.15E-15 ±23.3 | 1.16E-15 ±24.4 | 6.6E-01 |
| P duration V4 | 1.93E-15 ±18.5 | 9.07E-15 ±18.1 | 9.1E-01 |
| P duration V5 | -1.40E-15 ±16.9 | 7.26E-15 ±17.2 | 9.7E-01 |
| P duration V6 | 6.50E-16 ±16.6 | -7.73E-15 ±17.1 | 8.7E-01 |
| P amplitude I | -3.74E-15 ±28.4 | 6.79E-15 ±29.9 | 6.5E-01 |
| P amplitude II | -5.87E-15 ±41.1 | -2.85E-15 ±42.1 | 6.5E-01 |
| P amplitude V1 | -7.51E-16 ±59.7 | -5.11E-16 ±55.0 | 2.3E-01 |
| P amplitude V2 | 4.34E-15 ±45.8 | 4.84E-15 ±43.5 | 7.7E-01 |
| P amplitude V3 | 1.48E-15 ±21.9 | 1.24E-14 ±23.01 | 5.7E-01 |
| P amplitude V4 | -2.74E-15 ±19.4 | 3.51E-15 ±20.5 | 4.5E-01 |
| P amplitude V5 | 2.25E-15 ±18.9 | 7.05E-15 ±20.3 | 2.8E-01 |
| P amplitude V6 | -1.62E-15 ±19.03 | -2.68E-15 ±20.4 | 6.4E-01 |
| PR interval | 3.45E-15 ±16.2 | -7.14E-15 ±16.8 | 7.6E-01 |
| P axis | 3.37E-15 ±20.2 | 3.75E-15 ±20.7 | 8.4E-01 |
| P offset | -2.44E-15 ±9.5 | 1.02E-14 ±9.1 | 5.8E-01 |
| P onset | 1.51E-14 ±8.5 | 2.27E-15 ±9.1 | 8.1E-01 |
| Q area I | -5.61E-16 ±14.9 | 1.05E-15 ±14.2 | 1.2E-04 |
| Q area II | 1.78E-16 ±12.5 | 3.02E-15 ±12.1 | 6.1E-12 |
| Q area V5 | -1.91E-15 ±10.3 | 2.35E-15 ±10.2 | 1.4E-29 |
| Q area V6 | 3.75E-15 ±13.7 | 1.27E-15 ±12.8 | 1.7E-08 |
| Q duration I | -5.02E-16 ±8.1 | -5.75E-17 ±7.6 | 2.2E-04 |
| Q duration II | 1.84E-16 ±6.9 | 1.01E-15 ±7.05 | 2.3E-13 |
| Q duration V5 | 1.47E-15 ±6.6 | -2.30E-16 ±6.0 | 9.4E-21 |
| Q duration V6 | -1.04E-17 ±7.2 | -1.24E-15 ±6.8 | 6.7E-06 |
| Q amplitude I | 4.88E-16 ±25.6 | -2.78E-15 ±25.6 | 7.5E-05 |
| Q amplitude II | 1.78E-15 ±25.6 | 2.63E-15 ±26.2 | 2.6E-14 |
| Q amplitude V5 | 5.21E-15 ±24.9 | -2.73E-16 ±24.04 | 1.4E-21 |
| Q amplitude V6 | -1.10E-14 ±26.4 | 8.40E-16 ±25.3 | 4.0E-07 |
| QT interval | -6.61E-15 ±21.4 | -2.91E-14 ±22.2 | 8.8E-01 |
| Q offset | 1.20E-14 ±5.3 | -3.09E-15 ±5.1 | 8.4E-01 |
| Q onset | 1.79E-14 ±4.3 | -1.11E-15 ±4.4 | 9.5E-01 |
| QRS area I | 4.91E-15 ±293.2 | -6.90E-14 ±264.5 | 8.3E-01 |
| QRS area II | -4.29E-15 ±309.3 | -8.68E-14 ±292.4 | 2.7E-01 |
| QRS area V1 | 7.17E-15 ±429.2 | -1.13E-14 ±431.9 | 9.9E-01 |
| QRS area V2 | -3.24E-14 ±696.5 | 1.13E-14 ±711.3 | 6.2E-01 |
| QRS area V3 | 1.01E-14 ±814.3 | 8.89E-15 ±788.08 | 8.1E-01 |
| QRS area V4 | -3.10E-14 ±613.4 | 4.07E-15 ±612.6 | 8.0E-01 |
| QRS area V5 | 1.47E-13 ±461.6 | 1.15E-14 ±419.3 | 6.9E-01 |
| QRS area V6 | -3.45E-14 ±445.4 | 7.06E-14 ±410.1 | 7.0E-01 |
| QRS duration | -2.73E-15 ±8.3 | 5.09E-15 ±7.2 | 5.6E-01 |
| QRS interval I | -2.57E-16 ±4.2 | -1.13E-15 ±4.5 | 8.5E-01 |
| QRS interval II | 4.35E-15 ±5.7 | 1.04E-15 ±5.6 | 4.1E-01 |
| QRS interval V1 | 1.40E-15 ±7.4 | -1.42E-15 ±7.5 | 7.7E-01 |
| QRS interval V2 | -2.35E-15 ±9.8 | -3.77E-15 ±9.5 | 3.6E-01 |
| QRS interval V3 | -3.41E-15 ±11.2 | 2.71E-15 ±10.9 | 7.4E-01 |
| QRS interval V4 | -2.17E-15 ±7.6 | 8.18E-16 ±8.1 | 7.2E-01 |
| QRS interval V5 | -2.61E-15 ±5.5 | 6.25E-15 ±5.6 | 4.9E-01 |
| QRS interval V6 | -3.85E-15 ±5.3 | -1.85E-15 ±5.3 | 8.4E-01 |
| QTc calculation | 4.09E-14 ±18.6 | 1.21E-14 ±18.1 | 9.4E-01 |
| R area I | -1.43E-14 ±253.3 | 8.01E-16 ±238.9 | 5.7E-01 |
| R area II | 2.24E-14 ±288.01 | 6.60E-15 ±288.1 | 3.5E-01 |
| R area V1 | 9.81E-15 ±95.8 | -9.32E-15 ±97.8 | 6.9E-01 |
| R area V2 | 7.18E-15 ±233.8 | 2.34E-14 ±232.9 | 7.6E-01 |
| R area V3 | 3.37E-14 ±437.7 | -4.97E-14 ±405.2 | 4.1E-01 |
| R area V4 | -1.85E-13 ±452.2 | -1.20E-13 ±437.9 | 8.6E-01 |
| R area V5 | 2.95E-14 ±424.1 | -3.31E-15 ±386.8 | 6.3E-01 |
| R area V6 | 6.86E-14 ±389.8 | 1.63E-14 ±368.1 | 8.6E-01 |
| R duration I | 1.42E-16 ±14.9 | 7.49E-15 ±15.01 | 1.9E-01 |
| R duration II | 3.31E-15 ±14.04 | -5.64E-15 ±14.07 | 4.5E-01 |
| R duration V1 | -4.83E-16 ±7.8 | -2.50E-15 ±8.03 | 3.5E-01 |
| R duration V2 | 3.66E-15 ±10.2 | 5.95E-16 ±10.7 | 1.9E-01 |
| R duration V3 | -1.37E-15 ±9.4 | -2.71E-16 ±9.1 | 8.6E-01 |
| R duration V4 | 3.54E-15 ±8.9 | -2.33E-16 ±8.7 | 4.9E-01 |
| R duration V5 | 4.20E-16 ±9.7 | 1.31E-15 ±9.7 | 6.2E-01 |
| R duration V6 | -2.16E-15 ±12.4 | -1.30E-15 ±12.3 | 4.3E-01 |
| R amplitude I | 4.44E-14 ±178.7 | 3.99E-14 ±172.1 | 6.5E-01 |
| R amplitude II | 1.54E-14 ±220.5 | 3.33E-14 ±221.7 | 3.5E-01 |
| R amplitude V1 | -1.19E-14 ±104.1 | -1.50E-15 ±98.3 | 7.0E-01 |
| R amplitude V2 | 3.35E-14 ±228.8 | -1.46E-14 ±231.1 | 9.9E-01 |
| R amplitude V3 | -8.26E-14 ±342.9 | -5.42E-14 ±336.2 | 8.0E-01 |
| R amplitude V4 | -7.70E-14 ±397.3 | 1.33E-14 ±387.3 | 9.9E-01 |
| R amplitude V5 | -8.84E-15 ±381.01 | 6.26E-14 ±340.1 | 5.8E-01 |
| R amplitude V6 | 3.10E-14 ±335.6 | -3.30E-14 ±319.6 | 7.9E-01 |
| R axis | 4.41E-15 ±22.6 | -5.58E-16 ±21.8 | 4.8E-01 |
| S area I | 1.87E-15 ±26.4 | 1.98E-15 ±31.4 | 4.3E-22 |
| S area II | 6.88E-15 ±66.01 | -1.55E-14 ±61.8 | 3.4E-08 |
| S area V1 | 1.15E-13 ±435.8 | 4.78E-15 ±467.2 | 5.8E-01 |
| S area V2 | 1.08E-13 ±599.8 | 5.99E-14 ±654.9 | 8.5E-01 |
| S area V3 | -1.72E-14 ±485.1 | -5.88E-14 ±502.09 | 8.8E-01 |
| S area V4 | -2.23E-14 ±284.2 | -6.90E-14 ±289.02 | 6.8E-01 |
| S area V5 | -1.22E-14 ±177.9 | -1.08E-14 ±186.5 | 5.4E-01 |
| S area V6 | 8.53E-15 ±154.5 | -2.46E-15 ±164.5 | 5.5E-03 |
| S duration I | 3.62E-15 ±13.1 | -9.08E-16 ±11.5 | 1.1E-16 |
| S duration II | -1.33E-15 ±12.1 | -1.37E-15 ±11.1 | 7.4E-12 |
| S duration V1 | -3.31E-15 ±16.6 | -2.01E-15 ±17.1 | 7.2E-01 |
| S duration V2 | -1.30E-16 ±15.3 | -1.03E-15 ±16.4 | 6.8E-01 |
| S duration V3 | -5.67E-16 ±12.7 | -2.10E-15 ±12.1 | 6.8E-01 |
| S duration V4 | -2.92E-15 ±11.8 | 1.54E-17 ±10.7 | 8.4E-01 |
| S duration V5 | 2.09E-16 ±12.1 | -4.15E-16 ±9.9 | 8.9E-01 |
| S duration V6 | 3.26E-15 ±14.4 | -6.41E-16 ±13.2 | 9.9E-03 |
| S amplitude I | 7.93E-15 ±46.9 | -4.02E-15 ±41.9 | 3.9E-15 |
| S amplitude II | -1.03E-14 ±69.4 | 5.55E-15 ±67.3 | 3.4E-08 |
| S amplitude V1 | 3.26E-14 ±290.5 | -1.52E-14 ±296.9 | 3.6E-01 |
| S amplitude V2 | -1.04E-14 ±405.9 | -3.41E-14 ±437.1 | 8.1E-01 |
| S amplitude V3 | -9.17E-15 ±341.8 | 8.29E-15 ±337.8 | 7.4E-01 |
| S amplitude V4 | 9.23E-15 ±236.2 | 2.94E-15 ±236.7 | 8.3E-01 |
| S amplitude V5 | 1.00E-14 ±151.6 | 1.78E-14 ±169.6 | 4.1E-01 |
| S amplitude V6 | -4.16E-15 ±157.4 | -2.43E-15 ±159.8 | 2.4E-02 |
| T area I | -9.25E-14 ±524.8 | 6.98E-14 ±482.7 | 9.6E-01 |
| T area II | -6.38E-14 ±587.2 | 1.69E-14 ±584.3 | 5.8E-01 |
| T area V1 | 7.77E-15 ±866.7 | 2.70E-15 ±774.7 | 7.6E-01 |
| T area V2 | 7.73E-14 ±1356.7 | -1.10E-13 ±1292.7 | 3.4E-01 |
| T area V3 | 1.13E-13 ±1113.6 | 1.09E-13 ±1070.2 | 6.7E-01 |
| T area V4 | 1.77E-13 ±966.3 | 1.59E-13 ±885.6 | 6.2E-01 |
| T area V5 | -1.21E-14 ±814.7 | -2.04E-13 ±790.08 | 3.2E-01 |
| T area V6 | -8.57E-15 ±730.3 | -6.11E-14 ±700.02 | 4.5E-01 |
| T duration I | -2.13E-15 ±21.7 | 2.22E-15 ±21.4 | 4.0E-01 |
| T duration II | 3.68E-15 ±20.6 | -1.26E-14 ±20.5 | 9.6E-01 |
| T duration V1 | -6.51E-15 ±57.4 | -1.01E-14 ±57.9 | 9.0E-01 |
| T duration V2 | 1.29E-14 ±33.6 | 1.56E-14 ±33.8 | 7.6E-01 |
| T duration V3 | -7.89E-15 ±20.3 | -9.24E-15 ±21.1 | 9.2E-01 |
| T duration V4 | 1.34E-14 ±18.1 | 1.71E-14 ±19.8 | 9.7E-01 |
| T duration V5 | 7.58E-15 ±19.6 | -8.93E-15 ±19.4 | 5.8E-01 |
| T duration V6 | -8.09E-15 ±21.1 | -8.77E-15 ±22.1 | 3.9E-01 |
| T end I | 2.24E-15 ±28.2 | -1.26E-15 ±28.5 | 9.0E-01 |
| T end II | 3.08E-15 ±34.9 | -1.67E-15 ±35.8 | 7.3E-01 |
| T end V1 | 1.10E-15 ±33.6 | 4.83E-16 ±29.9 | 6.1E-01 |
| T end V2 | -2.20E-15 ±37.8 | 1.69E-16 ±35.1 | 7.0E-01 |
| T end V3 | 2.25E-15 ±36.3 | -2.45E-15 ±34.3 | 4.8E-01 |
| T end V4 | -1.48E-15 ±35.4 | 4.69E-15 ±30.6 | 5.8E-01 |
| T end V5 | -9.51E-16 ±30.08 | 1.32E-15 ±29.4 | 6.0E-01 |
| T end V6 | 1.55E-15 ±30.3 | -1.49E-15 ±27.2 | 5.6E-01 |
| T amplitude I | -2.49E-15 ±88.1 | -3.07E-15 ±78.1 | 9.9E-01 |
| T amplitude II | 6.36E-16 ±99.5 | -1.66E-14 ±102.2 | 3.1E-01 |
| T amplitude V1 | -2.21E-15 ±151.4 | 4.14E-15 ±136.8 | 9.6E-01 |
| T amplitude V2 | -1.90E-14 ±222.4 | 3.42E-15 ±214.5 | 4.1E-01 |
| T amplitude V3 | 7.57E-15 ±179.2 | -2.39E-15 ±175.8 | 6.3E-01 |
| T amplitude V4 | -3.10E-14 ±157.8 | 3.35E-14 ±150.9 | 4.4E-01 |
| T amplitude V5 | -3.29E-14 ±136.6 | 1.23E-15 ±130.7 | 1.7E-01 |
| T amplitude V6 | 3.15E-15 ±124.6 | 1.82E-14 ±122.7 | 5.5E-01 |
| T axis | 1.45E-15 ±21.5 | -7.62E-16 ±20.4 | 8.1E-01 |
| T offset | 1.20E-15 ±10.7 | -2.80E-14 ±11.3 | 8.2E-01 |
| Ventricular rate | 3.97E-15 ±6.8 | -1.10E-15 ±7.6 | 5.8E-01 |
| The results represent residuals acting as standardised change scores, which were calculated by regressing the ECG biomarker values from the repeat imaging visit on those from baseline visit. | | | |
